# The OsAGO2–*OsNAC300*–*OsNAP* module regulates leaf senescence in rice

**DOI:** 10.1111/jipb.13766

**Published:** 2024-08-22

**Authors:** Shaoyan Zheng, Junyu Chen, Ying He, Jingqin Lu, Hong Chen, Zipeng Liang, Junqi Zhang, Zhenlan Liu, Jing Li, Chuxiong Zhuang

**Affiliations:** ^1^ State Key Laboratory for Conservation and Utilization of Subtropical Agro‐bioresources, Guangdong Laboratory for Lingnan Modern Agriculture, College of Life Sciences South China Agricultural University Guangzhou 510642 China

**Keywords:** DNA methylation, leaf senescence, OsAGO2, *OsNAC300*, *OsNAP*

## Abstract

Leaves play a crucial role in the growth and development of rice (*Oryza sativa*) as sites for the production of photosynthesis. Early leaf senescence leads to substantial drops in rice yields. Whether and how DNA methylation regulates gene expression and affects leaf senescence remains elusive. Here, we demonstrate that mutations in rice *ARGONAUTE 2* (*OsAGO2*) lead to premature leaf senescence, with chloroplasts in *Osago2* having lower chlorophyll content and an abnormal thylakoid structure compared with those from wild‐type plants. We show that OsAGO2 associates with a 24‐nt microRNA and binds to the promoter region of *OsNAC300*, which causes DNA methylation and suppressed expression of *OsNAC300*. Overexpressing *OsNAC300* causes the similar premature leaf senescence as *Osago2* mutants and knocking out *OsNAC300* in the *Osago2* mutant background suppresses the early senescence of *Osago2* mutants. Based on yeast one‐hybrid, dual‐luciferase, and electrophoresis mobility shift assays, we propose that OsNAC300 directly regulates transcription of the key rice aging gene NAC‐like, activated by APETALA3/PISTILLATA (*OsNAP*) to control leaf senescence. Our results unravel a previously unknown epigenetic regulatory mechanism underlying leaf senescence in which OsAGO2–OsNAC300*–OsNAP* acts as a key regulatory module of leaf senescence to maintain leaf function.

## Graphical abstract

Rice ARGONAUTE 2 binds to the microRNA miR2863c to regulate the expression of the transcription factor gene *OsNAC300* through DNA methylation, ensuring the correct initiation of leaf senescence. The transcription factor OsNAP initiates the normal onset of senescence, directly or indirectly regulating the expression of senescence‐associated genes and chloroplast development.

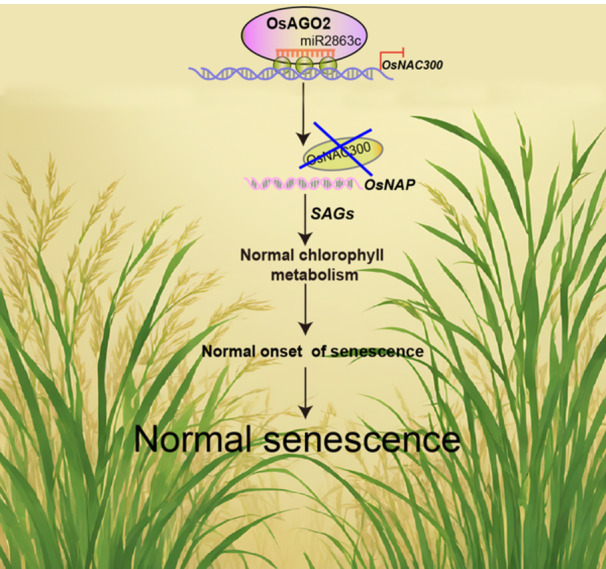

## INTRODUCTION

Rice (*Oryza sativa* L.) is an important cereal food crop worldwide, but leaf senescence limits rice yields in agricultural production. In the late stages of rice growth and development, premature leaf senescence shortens the window of active photosynthesis and grain‐filling, resulting in decreased dry biomass accumulation, which affects the development of rice grains, decreasing crop yields and quality (Schippers, 2015). At the same time, the natural aging of leaves promotes reallocation of photosynthates and nutrients from aging source tissues (leaves) to sink organs (grains), which greatly improves rice yield.

During aging, the color of leaves changes from green to yellow, reflecting chlorophyll degradation concomitant with disintegration of intracellular organelles, arrest of physiological metabolism, and changes in gene expression. In addition to chlorophyll and protein degradation, levels of reactive oxygen species (ROS) increase, culminating in lower overall chloroplast function during leaf aging (Lim et al., 2007; Woo et al., 2019). Most of the genes related to rice leaf senescence are involved in the regulation of chlorophyll degradation, ROS scavenging, carbon–nitrogen balance, and phytohormone responses, among other functions. Identification and characterization of such genes will help elucidate the regulatory mechanisms governing leaf senescence (Kong et al., 2006; Jiang et al., 2011; Chen et al., 2013; Rong et al., 2013; Yamatani et al., 2013; Liang et al., 2014; Yang et al., 2016a, 2016b; Bi et al., 2017).

Leaf senescence is regulated by a complex genetic network that includes many members of the large and plant‐specific NAC (for NO APICAL MERISTEM, ATAF2, CUP‐SHAPED COTYLEDON) family of transcription factors (Buchanan‐Wollaston et al., 2005; Gregersen and Holm, 2007; Balazadeh et al., 2008; Christiansen and Gregersen, 2014; Cao et al., 2023). NAC‐type transcription factors primarily function by regulating the expression of target genes or by interacting with other transcription factors (Sakuraba et al., 2015; Kim et al., 2018). In Arabidopsis (*Arabidopsis thaliana*), ANAC002 (also named ATAF1), ANAC016, ANAC019, ANAC029 (also named NAC‐like ACTIVATED BY APETALA3/PISTILLATA (NAP)), ANAC032, ANAC046, ANAC055, ANAC072 (also named RESPONSIVE TO DESICCATION 26 (RD26)), ANAC087, and ANAC092 (also named ORESARA 1 (ORE1)) are positive regulators of leaf senescence (Kim et al., 2009; Takasaki et al., 2015; Oda‐Yamamizo et al., 2016). Arabidopsis NAP is an important regulator of senescence that controls the expression of *SENESCENCE‐ASSOCIATED GENE 113* (*SAG113*), a direct target gene of NAP that encodes a protein phosphatase 2C member. Expression of *NAP* and *SAG113* is induced by leaf senescence and abscisic acid (ABA), modulating stomatal movement and water loss and mediating leaf senescence (Guo and Gan, 2006; Zhang and Gan, 2012). More than 150 NAC‐type transcription factors have been identified in rice, some of which are also involved in controlling leaf senescence. Many NAC family members are induced by upstream senescence signals from the degradation of proteins, carbohydrates, lipids, and chlorophyll while regulating the expression of downstream senescence effector genes such as *OsNAP* (Kim et al., 2016; Cao et al., 2023). *OsNAP*, similar to Arabidopsis *NAP*, is highly expressed in senescing tissues and encodes a protein that directly regulates the response of *SAG*s to ABA treatment (Nuruzzaman et al., 2010; Kim et al., 2018). Overexpression of *OsNAP* in rice produces an early leaf senescence phenotype, while knocking down *OsNAP* transcript levels via RNA interference (RNAi) results in delayed leaf senescence, making *OsNAP* a key marker gene for leaf senescence onset (Liang et al., 2014). Another NAC member of rice, OsNAC2, which directly activates the expression of the chlorophyll degradation genes *STAY‐GREEN* (*OsSGR*) and *NON‐YELLOW COLORING 3* (*OsNYC3*), upregulates the expression of the ABA biosynthesis genes and downregulates the expression of the ABA catabolism genes, leading to an increase in ABA levels and promoting leaf senescence (Mao et al., 2017).

Epigenetic regulation also appears to play a substantial role in leaf senescence (Ostrowska‐Mazurek et al., 2020; Yuan et al., 2020). Epigenetic mechanisms in eukaryotes result in phenotypic or gene expression changes without altering DNA sequences. These mechanisms include regulation through small RNA pathways, post‐translational modifications of histones, chromatin remodeling, and DNA methylation, among others (Pandey et al., 2002; Ueda and Seki, 2020; Ren et al., 2022). In plants, DNA methylation is mediated through the RNA‐directed DNA methylation (RdDM) pathway, which involves small interfering RNAs (siRNAs) and scaffold RNAs in addition to an array of proteins such as ARGONAUTE (AGO) proteins (Satyaki and Gehring, 2017). The canonical RdDM pathway, which is mediated by Pol IV‐dependent 24‐nt siRNA, and binds to specific AGO proteins to form RNA‐induced silencing complexes (RISCs) and recognizes transcripts through base‐complementary pairing way, resulting in messenger RNA (mRNA) cleavage, translational repression, and DNA methylation (Wu et al., 2010; Gallego‐Bartolomé, 2020). Arabidopsis AGO2 recruits miR393b* and modulates the secretion of pathogenesis‐related proteins to regulate plant immunity (Zhang et al., 2011). A 24‐nt non‐canonical long miRNA, miR820.2, is loaded into AGO4 to direct DNA methylation at an *OsDRM2* target site via the non‐canonical RdDM pathway (Wu et al., 2009). There are still many unexplored issues regarding the role of small RNA‐mediated epigenetic modifications in maintaining genomic stability and regulating gene expression.

Altering the methylation levels of the promoters or coding regions of genes influences the binding capacity of transcription factors to regulatory sequences, thus regulating gene expression. During leaf senescence, DNA methylation tends to decrease (Ogneva et al., 2016; Lang et al., 2017; Yuan et al., 2020). Cytosines undergo methylation in one of three contexts, namely, CG, CHG, and CHH (where H represents A, T, or C) methylation. Changes in DNA methylation levels at these positions modulate the transcription of downstream senescence‐related genes, thereby altering leaf senescence (Kinoshita et al., 2007; He et al., 2018; Zicola et al., 2019; Yuan et al., 2020). In loss‐of‐function mutants for the DNA demethylase DML3, the promoter regions of SAGs exhibit high levels of methylation, leading to decreased transcription levels and delayed leaf senescence (Yuan et al., 2020). These findings demonstrate that DNA methylation is associated with leaf senescence.

We previously discovered that rice ARGONAUTE 2 (OsAGO2) regulates DNA methylation levels at the *HEXOKINASE 1* (*OsHXK1*) promoter, controlling its expression and thus regulating rice pollen development (Zheng et al., 2019). Here, we demonstrate that knocking down the expression of *OsAGO2* is associated with premature leaf senescence during the heading to grain‐filling stage. Transcriptomic, DNA methylation, and RNA immunoprecipitation (RIP) analyses revealed that OsAGO2 binds to a 24‐nt miRNA to regulate leaf senescence by mediating the methylation level along the promoter region of the NAC transcription factor gene *OsNAC300*, thereby suppressing its expression. Overexpression of *OsNAC300* leads to early leaf senescence. Yeast one‐hybrid (Y1H) and electrophoretic mobility shift assays (EMSAs) indicated that OsNAC300 promotes leaf senescence by binding to and activating the expression of the key senescence gene *OsNAP*. Our results reveal a previously uncharacterized pathway whereby OsAGO2 regulates leaf senescence through DNA methylation guided by miRNA.

## MATERIALS AND METHODS

### Plant materials and growth conditions

Seeds of the wild‐type (WT) rice (*Oryza sativa* ssp. *japonica* cv. Zhonghua 11 (ZH11)) used in this study have been deposited under the stock number ZH11 (84‐213) at the China Rice Data Center (https://www.ricedata.cn). All transgenic materials and WT were planted in a greenhouse at South China Agricultural University, Guangzhou, China. The mutant lines *Osago2‐1* and *Osago2‐2* obtained from the ZH11 background by clustered regularly interspaced small palindromic repeats (CRISPR)/CRISPR‐associated nuclease 9 (Cas9) method, the *OsAGO2* overexpression (OE) plants harboring an Flag‐*OsAGO2* transgene, *AGO2*‐OE1 and *AGO2*‐OE2 as described by Zheng et al. (2019), the *OsNAC300* OE lines *NAC300*‐OE1 and *NAC300*‐OE2, as well as the mutant lines *Osnac300‐1* and *Osnac300‐2*, which were identified by reverse transcription quantitative polymerase chain reaction (RT‐qPCR) and Sanger sequencing.

### Measurements of agronomic traits and characterization of phenotypes

Plants were photographed using a digital camera (Canon 750D; Tokyo, Japan) at the mature stage. Plant tissues at different developmental stages were collected according to plant development and morphology. Developmental stage was verified by examination of semi‐thin sections as described by Zheng et al. (2019). Transmission electron microscopy (TEM) observations were performed as described by Zheng et al. (2019). Briefly, the leaves from WT and mutant lines at the flowering stage were cut into approximately 0.1–0.2 cm segments and immediately placed in 3 mL of 4% (w/v) paraformaldehyde with 2.5% (w/v) glutaraldehyde fixative. Samples in fixative were then vacuum infiltrated for approximately 20 min and kept at 4°C overnight. The samples were treated with 1% (w/v) OsO_4_ in 0.1 mol/L phosphate buffer (pH 7.2) for 1–2 h. After washing three times in 0.1 mol/L phosphate buffer (pH 7.2) for 15 min each, the samples were dehydrated using a graded ethanol series before being transferred into Eponate 12 resin mixture (#18010; Ted Pella, Redding, California, USA) overnight. The specimens were then placed in capsules with embedding medium and heated to 60°C overnight. To determine chloroplast and thylakoid morphology, ultrathin sections (90 nm) were prepared using an EM UC7 ultramicrotome (Leica Microsystems; Wetzlar, Germany), collected on copper grids, and stained with uranyl acetate and lead citrate. The stained sections were examined and photographed under a Philips FEI Tecnai 12 TEM (Hillsborough, Oregon, USA). Traits of *NAC300*‐OE1 and *NAC300*‐OE2 OE plants were measured in the T3 and T4 generations. The method for determining the content of total chlorophyll and hydrogen peroxide in leaves were performed as per Zheng et al. (2021). Student's *t*‐test was used for statistical analysis. The asterisks represent significant differences at ***P < 0.001, **P < 0.01, and *P < 0.05.

### Reverse transcription qPCR assay and expression analysis

Total RNA was isolated from rice tissues using the Trizol reagent (GenStar, Beijing, China). The complementary DNA (cDNA) was synthesized with a HiScript III 1st Strand cDNA Synthesis Kit (Vazyme, Nanjing, China). The qPCR was performed by the RealStar Green Fast Mixture (GenStar) using the qTOWER^3G^ Real‐Time PCR Detection System (Analytik Jena, Germany), with three technical replicates per biological sample with *OsACTIN1* as an internal reference gene (Zheng et al., 2021). The primer sequences used for RT‐qPCR analysis are listed in Table S5.

### Phylogenetic analysis

Phylogenetic analysis of NAC proteins from rice and *Arabidopsis thaliana* was performed using the maximum‐likelihood method. NAC proteins regulating senescence were identified through the National Center for Biotechnology Information (NCBI) website (https://www.ncbi.nlm.nih.gov/). The resulting proteins containing No Apical Meristem (NAM) and non‐NAM domains in rice and other species were aligned using ClustalW‐Align in MEGA11. The domains of each NAC homologous protein were identified using the Conserved Domains Database tool of NCBI website (https://www.ncbi.nlm.nih.gov/cdd). The extent of sequence similarity between OsNAC300 and homologous proteins was determined using the CLUSTAL‐OMEGA website (https://www.ebi.ac.uk/Tools/msa/clustalo/). Accession numbers of proteins used in the phylogenetic analysis are listed in Table S3.

### The TUNEL assay

The TUNEL (terminal deoxynucleotidyl transferase‐mediated dUTP nick end‐labeling) assay was performed using a TUNEL kit (DeadEnd Fluorometric TUNEL system, G3250; Promega, Madison, WI, USA) according to the manufacturer's instructions and performed as Zheng et al. previously described (2019, 2022). Flag leaves at flowering stage were collected from ZH11, *Osago2‐1*, *Osago2‐2* plants. The samples were analyzed under a LSM510 confocal laser scanning microscope (Zeiss). Propylene iodide staining uses red fluorescence to represent the background, while TUNEL‐positive staining uses yellow to green fluorescence from the overlays between green fluorescein and propylene iodide signals to represent it. All photos were taken with the same settings.

### Dual‐luciferase transient expression assay

The binding activity of OsNAC300 to the *OsNAP* promoter was monitored using a dual‐luciferase transient expression system in protoplasts prepared from rice leaf sheaths as previously described (Chen et al., 2021). The whole *OsNAP* promoter and the fragments (P1–P4) were cloned into the double reporter vector pGreenII 0800‐LUC, while the full‐length coding sequence of *OsNAC300* was inserted into the pGreenII 62‐SK vector to generate an effector construct. The resulting reporter and effector plasmids were co‐transfected into rice leaf sheath protoplasts. The activity of firefly luciferase (LUC) and *Renilla* luciferase (REN) was measured using dual‐luciferase reagents (Promega). Results are expressed as a LUC/REN ratio, using the *35S:GFP* effector construct as an internal control. Each experiment was performed with more than three biological repeats.

### Electrophoretic mobility shift assay

Based on the dual‐luciferase reporter gene experiments and the DNA affinity purification followed by sequencing (DAP‐seq) results, we synthesized the probes of the *OsNAP* promoter region for the EMSA experiment. The EMSA probes were biotin‐labeled following the instructions of the EMSA DNA Labeling Kit (Thermo Fisher Scientific). The EMSA experimental procedures were carried out with the purified recombinant HIS‐OsNAC300 at 24°C for 30 min as per the LightShift Chemiluminescent EMSA Kit instructions (Thermo Fisher Scientific).

### Yeast one‐hybrid assay

Assays were performed according to the manufacturer's instructions (Cat. # 630491; Clontech Laboratories, Inc., a Takara Bio Company). Full‐length *OsNAC300* cDNA was cloned into the pGADT7 vector, and the promoter region of *OsNAP* was cloned into the pAbAi vector. The resulting constructs or corresponding empty vectors were co‐transformed into the yeast (*Saccharomyces cerevisiae*) strain Y1H Gold, and colonies of the corresponding transformants were incubated at 30°C on synthetic defined (SD)/−Ura and SD/−Leu medium with 100 ng/mL Aureobasidin A (AbA) for 2–3 d. Absence of Leu in the selective media is required for maintaining the bait and prey plasmids, while AbA in the selective media is crucial for selecting one‐hybrid interactions. Primers for these constructs are listed in Table S5.

### RNA sequencing and RNA analysis

Flag leaves at flowering stage were collected from ZH11, *Osago2‐1*, *Osago2‐2*, *NAC300*‐OE1, and *NAC300*‐OE2 plants grown under normal field conditions for transcriptome analysis. The RNA sequencing and bioinformatic analysis were performed by the Beijing Genomics Institution (BGI, Shenzhen, China). Differential expression analysis between ZH11 and *Osago2* plants, and WT and *NAC300*‐OE plants, was performed using the output from Significant Analysis of EBseq. A false discovery rate of 5% and a minimum fold change (FC) of 1 were used as thresholds for significant differential expression. Gene functions were annotated using the NCBI (ncbi.nlm.nih.gov), Pfam (https://pfam.xfam.org/), and Swiss‐Prot (https://www.uniprot.org/contact) databases. All differentially expressed genes (DEGs) in *Osago2* plants versus the WT, and *NAC300*‐OE plants versus the WT, are listed in Table S4.

### Chromatin immunoprecipitation‐qPCR assays

Chromatin immunoprecipitation (ChIP) assays were performed as previously described (Zheng et al., 2019; Lu et al., 2024). Briefly, flag leaves (2–4 g each) from WT and Flag‐*OsAGO2* transgene (*AGO2*‐OE) (in the ZH11 background) plants at the heading stage were crosslinked in 1% (w/v) formaldehyde under a vacuum for 15 min. The chromatin was extracted and fragmented by sonication (30 s on, 30 s off, 25 cycles). The WT and Flag‐*OsAGO2* plant chromatins were immunoprecipitated using an anti‐Flag antibody (Cat#: F3165; Sigma‐Aldrich) or anti‐rabbit immunoglobulin G (IgG: the negative control, Cat#: 14708; Cell Signaling Technology). Dynabeads protein A/G was added to the fragmented chromatin, followed by incubation at more than 6 h at 4°C. The purified chromatin DNA was subjected to qPCR using primers listed in Table S5. Each reaction was performed in triplicate. The difference between the *C*_*t*_ of the WT and *AGO2*‐OE samples was calculated to determine the relative enrichment of the upstream fragment as previously described (Zheng et al., 2019; Lu et al., 2024).

### RNA immunoprecipitation and analysis

RNA immunoprecipitation was performed as previously described (Carbonell, 2017). In brief, 3–4 g of leaf tissues from 14 d‐old seedlings were collected. Plant materials were ground to powder in liquid nitrogen and resuspended in protein extraction buffer (50 mmol/L Tris‐HCl, pH 7.4, 2.5 mmol/L MgCl_2_, 100 mmol/L KCl, 5 mmol/L MgCl_2_, 0.1% (v/v) Nonidet P‐40, 5 mmol/L dithiothreitol (DTT), 50 U/mL RNase inhibitor, and protease inhibitor cocktail (K1010; APExBIO)). The homogenate was mixed for 10 min and centrifuged at 4°C for 10 min at 8,000 *g*. The supernatant was transferred to a new tube, and anti‐Flag antibody (Cat#: F3165; Sigma‐Aldrich) or a negative control (anti‐rabbit IgG, Cat#: 14708; Cell Signaling Technology) were added into the tube. Tubes were incubated at 4°C for 3–4 h before the beads (Protein A/G agarose; Sigma‐Aldrich) were collected by centrifugation at 100 *g* for 30 s at 4°C. The beads were washed three times with protein washing buffer (20 mmol/L Tris‐HCl, pH 7.5, 300 mmol/L NaCl, 5 mmol/L MgCl_2_, 0.5% (v/v) Triton X‐100, 5 mmol/L DTT, and protease inhibitor cocktail (K1010; APExBIO)). The RNA from Flag‐AGO2 RIP was isolated by Trizol reagent and used in small RNA (sRNA) library construction, Illumina sequencing, and RT‐qPCR assays.

## RESULTS

### Loss of OsAGO2 function results in premature leaf senescence

**Figure 1:**
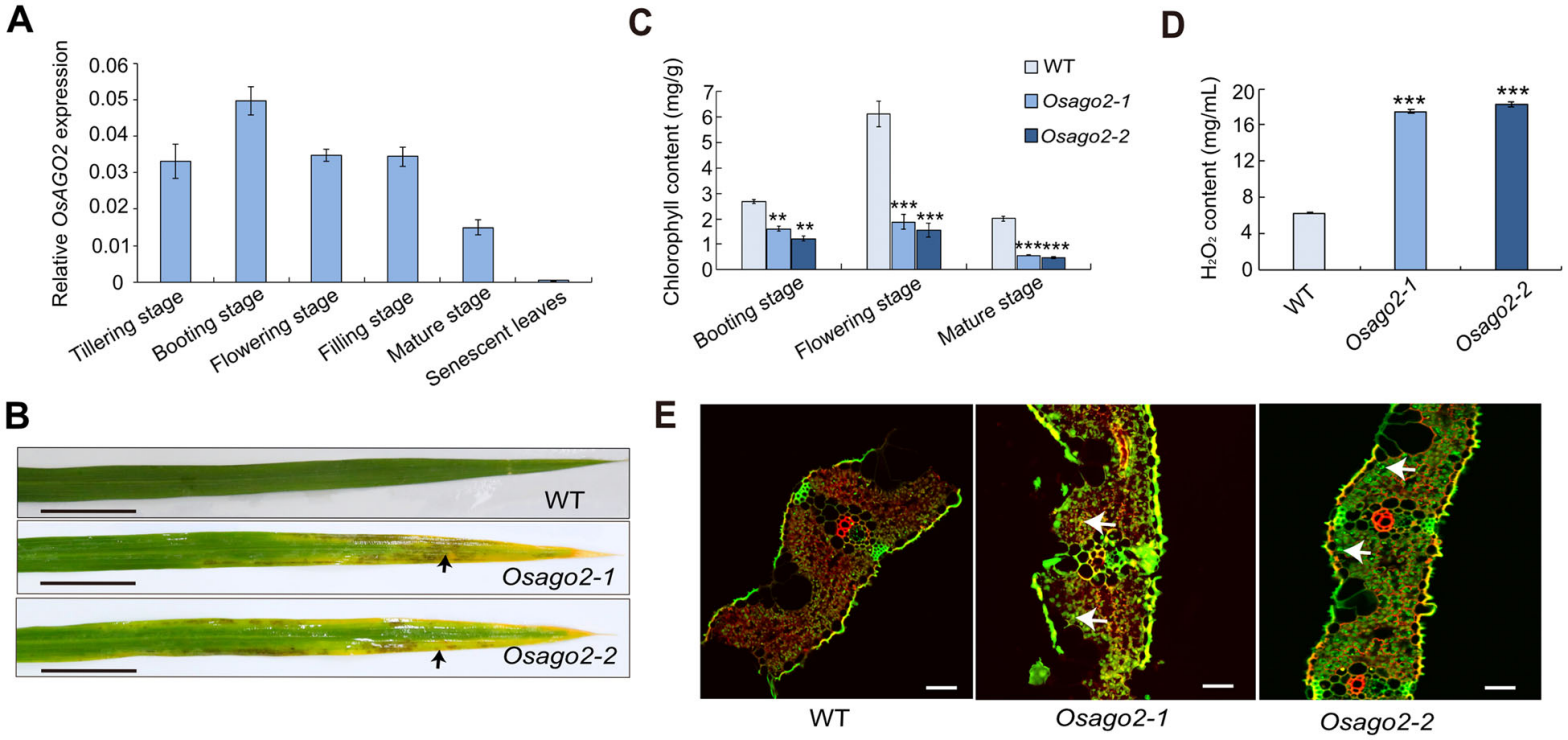
***Osago2*** **edited mutants show early leaf senescence** **(A)** Relative expression of *OsAGO2* at different development stages of leaf from tillering stage to senescent leaves in wild‐type (WT). All data are presented as means ± *SD* of three independent replicates. **(B)** Representative flag leaves from WT, *Osago2‐1*, and *Osago2‐2* plants at the flowering stage. Bars = 4 cm. **(C)** Total chlorophyll contents of WT, *Osago2‐1*, and *Osago2‐2* plants at the booting to mature stages. All data are presented as means ± *SD* of three independent replicates. **0.001 < *P* < 0.01. ***P < 0.001. *P*‐values were determined by Student's *t*‐test. **(D)** H_2_O_2_ contents in the leaves of WT, *Osago2‐1*, and *Osago2‐2* plants at the flowering stage. ***P < 0.001. *P*‐values were determined by Student's *t*‐test. All data are presented as means ± *SD* from three independent replicates. **(E)** DNA fragment signals from WT and *OsAGO2* mutant plants at the flowering stage leaves. Red fluorescence from the staining of leaves with propidium iodide (PI) was visualized by confocal laser scanning microscopy; images show overlays of green fluorescence from TUNEL (terminal deoxynucleotidyl transferase‐mediated dUTP nick end‐labeling) signals and red fluorescence from PI staining. The white arrows showed the abnormal DNA fragment signals of green fluorescence. Bars = 20 µm.

To study how OsAGO2 functions in the rice leaves, we performed RT‐qPCR to analyze the expression patterns of *OsAGO2* in the leaves at the different stages of WT; the results showed that *OsAGO2* is expressed in leaves at various stages and is highly expressed in leaves during the booting stage and the lowest expression was detected in senescent leaves (Figure 1A). Furthermore, we observed premature senescence in the leaves of *Osago2* mutant plants (named *Osago2‐1* and *Osago2‐2*) obtained from the ZH11 (*Oryza sativa* ssp. *japonica* cv. Zhonghua 11) background by the CRISPR/Cas 9 method (Figure S1A). These results supported the postulation that OsAGO2 might be involved in maintaining the leaf function of rice. We thus assessed the function of OsAGO2 in leaf of rice. Phenotypically, *Osago2‐1* and *Osago2‐2* plants displayed a noticeable withered and yellow leaf senescence phenotype compared with WT plants at the flowering stage (Figure 1B). Consistent with their visible premature senescence phenotypes, *Osago2‐1* and *Osago2‐2* plants showed decreased chlorophyll contents from the booting to mature stages and higher accumulation of ROS at the flowering stage (Figure 1C, D, S1B, S1C). To determine the effects of altered OsAGO2 function on leaf programmed cell death (PCD) in *Osago2* senescent leaves, the terminal deoxynucleotidyl TUNEL assay was performed due to the early senescent leaf phenotype in *Osago2* mutants. We detected more prominent TUNEL‐positive signals at the flowering stage in leaves of *Osago2‐1* and *Osago2‐2* plants than in leaves of WT plants, in which we rarely observed a TUNEL signal for nucleic acid fragmentation (Figure 1E). This result indicates that PCD occurred earlier in *Osago2* mutant plants than in WT plants. We conclude that loss of OsAGO2 function is responsible for the premature leaf senescence phenotype observed in *Osago2* mutants.

### Loss of OsAGO2 function causes premature leaf senescence by affecting the chloroplast function

**Figure 2:**
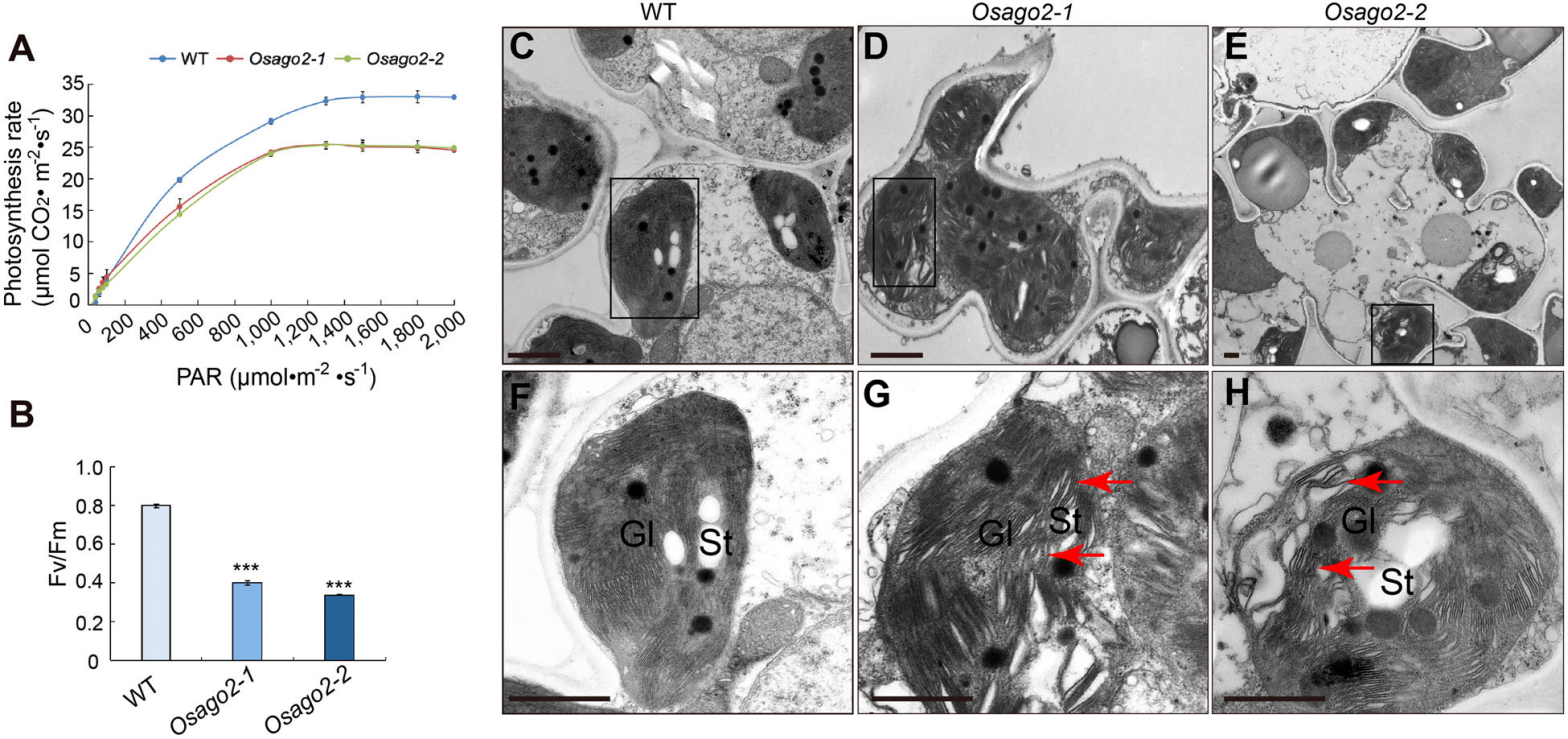
***Osago2*** **mutants have impaired photosynthesis resulting from altered chloroplast ultrastructure** **(A)** Photosynthesis rates of wild‐type (WT), *Osago2‐1*, and *Osago2‐2* plants at the flowering stage. All data are presented as means ± *SD* of three independent replicates. PAR, photosynthetically active radiation. **(B)** *Fv/Fm* (variable fluorescence/fluorescence maximum) values of WT, *Osago2‐1*, and *Osago2‐2* plants at the flowering stage. All data are presented as means ± *SD* of three independent replicates. ***P < 0.001. *P*‐values were determined by Student's *t*‐test. **(C–H)** Transmission electron microscopy (TEM) analysis of leaf sections from WT, *Osago2‐1*, and *Osago2‐2* plants at the flowering stage. **(F** ***–*** **H)** are the enlarged images in black box from **(C** ***–*** **E)**. The red arrows showed the abnormal grana lamellae. GL, grana lamellae; St, starch grain. Bars = 1 µm.

Leaves are the main organs of photosynthesis; to explore the consequences of loss of OsAGO2 function, we measured gas exchange and examined photosynthesis fluorescent parameters of all plants. *Osago2‐1* and *Osago2‐2* plants showed aberrant F_v_/F_m_ (variable fluorescence/fluorescence maximum) values which reflect the potential maximum photosynthetic capacity of plants, only reaching about 50% of WT levels, as well as lower photosynthesis rates compared with WT plants under normal growth conditions (Figures 2A, B, S1D). These results suggest that the premature leaf senescence observed in *Osago2* mutants is accompanied by lower photosynthetic activity or low utilization efficiency of light. As flag leaves of *Osago2‐1* and *Osago2‐2* accumulated less total chlorophyll than those of WT plants from the booting to mature stages (Figure 1C), we examined the ultrastructure of WT, *Osago2‐1*, and *Osago2‐2* chloroplasts using TEM at the flowering stage. In WT leaves, chloroplast structure was regular, with well stacked and clearly visible grana lamellae (Figure 2C, F). By contrast, chloroplasts of *Osago2‐1* and *Osago2‐2* flag leaves contained abnormal and loose thylakoid grana lamellae, with degraded chloroplast structures (Figure 2D, G, E, H). These results indicate that the lower chlorophyll content and early senescence leaf phenotypes of the *Osago2* mutants are accompanied by chloroplast degradation, suggesting a vital role for OsAGO2 in maintaining the function of chloroplasts.

### OsAGO2 binds to a 24‐nt miRNA and represses *OsNAC300* transcription through DNA methylation

To reveal the molecular basis for the regulation of leaf senescence by OsAGO2, we performed deep sequencing (RNA‐seq) to profile the transcriptomes of leaves from WT plants and *Osago2* mutants at the flowering stage. We identified 3,393 DEGs, with 1,968 genes being significantly upregulated (|log_2_ FC|≥ 1 and *Q*‐value < 0.05) and 1,425 genes being downregulated (|log_2_ FC|≤ −1 and *Q*‐value < 0.05) in *Osago2* mutants compared with WT plants (Figure S2A). Consistent with the observation of premature leaf senescence, expression of *SAGs* (senescence‐associated genes) was increased in *Osago2* mutant plants compared with WT plants (Figure S2B‐I). Previous genetic and molecular studies have suggested that multiple NACs mediate leaf senescence (Gregersen and Holm, 2007; Mao et al., 2017). In rice and Arabidopsis, NACs play a role in mediating leaf senescence by activating the expression of *SAG*s, *CCG*s (chlorophyll catabolism genes), and other genes (Liang et al., 2014; Mao et al., 2017). We thus examined the list of DEGs between *Osago2* and WT plants and identified 17 upregulated NAC family genes in the *Osago2* mutants, most of which were associated with leaf senescence in previous reports (Table S1). Accordingly, we conducted whole‐genome bisulfite sequencing (WGBS) to profile the methylomes of WT and *Osago2* plants before flowering stage. When examining the methylation levels of differentially methylated regions (DMRs) in *Osago2* mutants in all sequence contexts (CG, CHG, and CHH), we observed significantly lower methylation in the *Osago2* mutant background than in the WT background (Figure S3A). The average methylation levels in the CG context of *Osago2* mutants decreased during leaf senescence, especially during the promoter regions (Figure S3B). In contrast to the WT where hypemethylation occurred during leaf senescence, fewer hypemethylated DMRs were observed in the *Osago2* mutant in the CG/CHG/CHH contexts, while the opposite was true for hypomethylation DMRs (Figure S3C). Analysis of the changes in global DNA methylation in the WT and *Osago2* plants, within these NAC transcription factor genes, one, namely *OsNAC300*, exhibited a noticeable change in the methylation level along the entire of its promoter region (Figure S3D).

**Figure 3:**
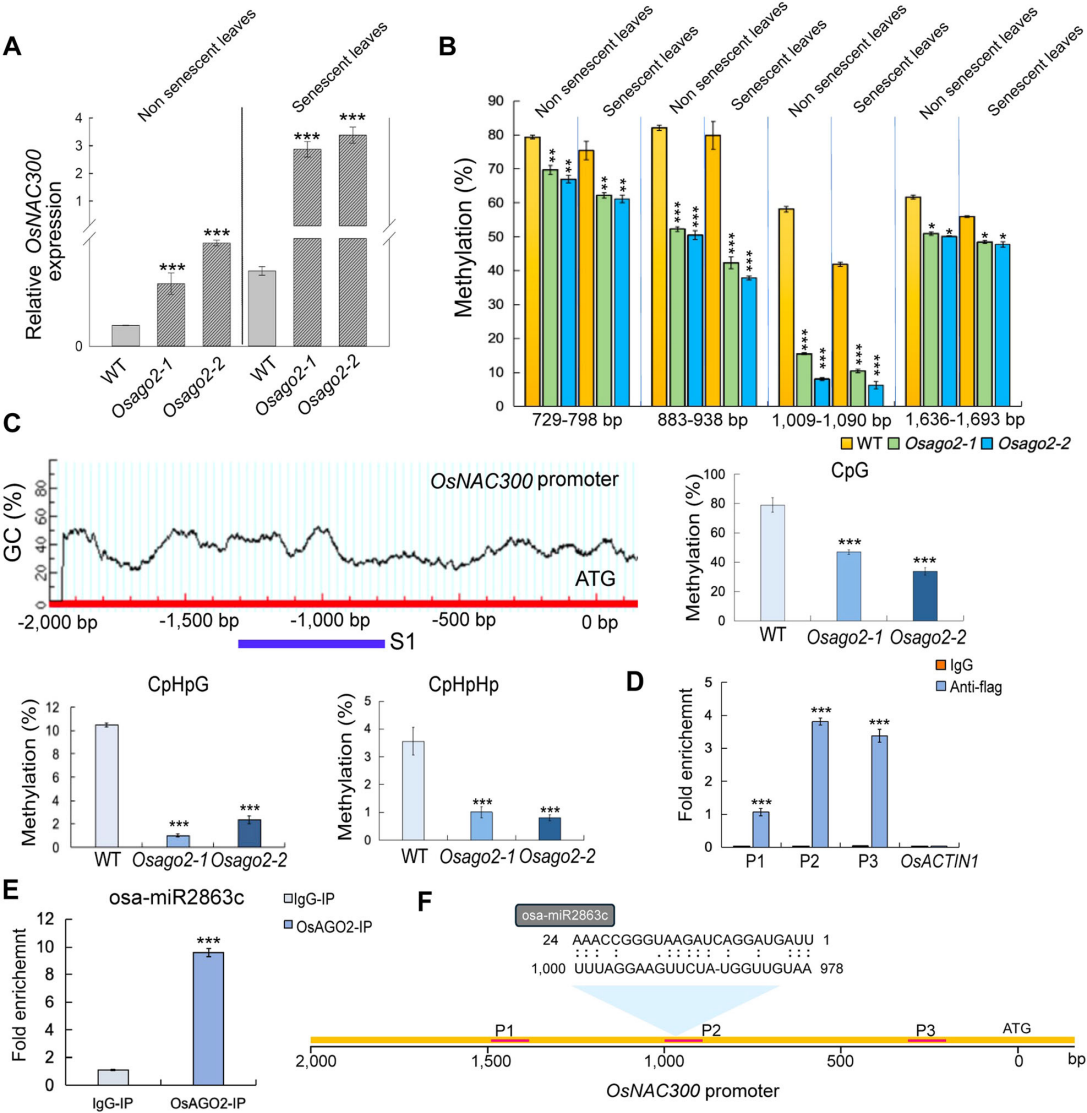
***Oryza sativa*** **ARGONAUTE 2 (OsAGO2) directly targets the** ***OsNAC300*** **promoter to modulate its DNA methylation pattern and regulate early leaf senescence in rice** **(A)** Relative *OsNAC300* transcript levels in the leaves of wild‐type (WT), *Osago2‐1*, and *Osago2‐2* plants at the non‐senescent stage (tillering stage with no visible sign of yellowing of the WT, *Osago2‐1* and *Osago2‐2* plants) and senescent stage (flowering stage with visible sign of yellowing of *Osago2‐1* and *Osago2‐2* plant). All data are presented as means ± *SD* from three independent replicates. ***P < 0.001. *P*‐values were determined by Student's *t*‐test. **(B)** Percentage of methylation at individual cytosine sites, as determined by pyrosequencing, along the region of the *OsNAC300* promoter in WT, *Osago2‐1*, and *Osago2‐2* plants on the same two stages shown in **(A)**. All data are presented as means ± *SD* of three independent replicates. *0.01 < *P* < 0.05, **0.001 < *P* < 0.01, ***P < 0.001. *P*‐values were determined by Student's *t*‐test. **(C)** DNA methylation analysis along the *OsNAC300* promoter region in WT, *Osago2‐1*, and *Osago2‐2* plants by bisulfite sequencing. Sequencing data were analyzed with Kismeth software. *0.01 < *P* < 0.05, ***P < 0.001. *P*‐values were determined by Student's *t*‐test. **(D)** Chromatin immunoprecipitation quantitative polymerase chain reaction (ChIP‐qPCR) analysis of OsAGO2 binding to the *OsNAC300* promoter in the leaves of transgenic rice plants harboring the *Ubipro:Flag‐AGO2* construct, using an anti‐Flag antibody. Negative control, anti‐immunoglobulin G (anti‐IgG: normal rabbit IgG antibody). P1–P3, three different sequences in the *OsNAC300* promoter region indicated in **(F)**. Data were normalized to input chromatin. The IgG antibody was used as the negative control, and *OsACTIN1* was used as the reference. Values are means ± *SD* (*n* = 3 biological repeats). ***P < 0.001 (Student's *t*‐test). **(E)** RNA immunoprecipitation (RIP)‐qPCR showing that OsAGO2 binds to miR2863c. Anti‐Flag was used for the RIP experiment. Data are means ± *SD*. ***P < 0.001 according to Student's *t*‐test compared with the IgG antibody (used as the negative control). The experiment was repeated three times with similar results. **(F)** The diagram of binding regions of OsAGO2 to the *OsNAC300* promoter region determined by ChIP‐qPCR and RIP‐qPCR analysis.

Moreover, compared with non‐senescent leaves, *OsNAC300* is more significantly expressed in *Osago2* senescent leaves (Figure 3A). We suggest that *OsNAC300* might be a target of OsAGO2. Further, the analysis of the *OsNAC300* genomic sequence revealed it has a GC‐rich promoter region, so we hypothesized that epigenetic regulation, particularly DNA methylation, might be responsible for the relationship between OsAGO2 function and *OsNAC300* expression levels. To test this possibility, we analyzed the DNA methylation level in the *OsNAC300* promoter region in non‐senescent and early senescence leaves of WT and *Osago2* plants by pyrosequencing. The result showed that the *OsNAC300* promoter (729–1,693‐bp) regions of the two *Osago2‐1* and *Osago2‐2* mutants showed significantly lower methylation levels than those in the WT. We also observed the significantly lower levels (<50%) of CG, CHG, and CHH methylation in the *OsNAC300* promoter (883–938 and 1,009–1,090 bp) region in senescent leaves of *Osago2‐1* and *Osago2‐2* plants than in those of WT plants (Figure 3B). Compared with non‐senescent leaves, the methylation levels of *OsNAC300* decreased more significantly in the early senescent leaves of the *Osago2* mutants (Figure 3B).

We also assessed the methylation pattern over the entire length of the 2‐kb region of the *OsNAC300* promoter at all cytosine sites. Specifically, *Osago2* showed reduced CG, CHG, and CHH methylation levels, while WT exhibited increased methylation (Figure 3C). We performed ChIP‐qPCR assays to validate the binding of OsAGO2 to the *OsNAC300* promoter, targeting specific regions in the *OsNAC300* promoter. Using the *OsAGO2* OE lines harboring an Flag‐*OsAGO2* transgene (*AGO2*‐OE), we observed enrichment for three DNA sequences (P1–P3) in the chromatin immunoprecipitated by OsAGO2 using an anti‐Flag antibody. As an important control, we detected no enrichment for the *OsACTIN1* locus, which lacks OsAGO2‐binding sequences (Figure 3D).

OsAGO2 might promote DNA methylation through sRNA guidance due to AGO being a key component in the rice gene‐silencing machinery (Liu and Chen, 2016). To identify which sRNA was associated with OsAGO2 and promoted *OsNAC300* promoter DNA methylation, we also performed a RIP experiment using an anti‐Flag antibody to precipitate Flag‐tagged OsAGO2 from *AGO2*‐OE transgenic lines. Notably, sRNA deep sequencing results indicated that a 24‐nt miR2863c was significantly enriched in the OsAGO2‐IP fraction from *AGO2*‐OE transgenic samples (Table S2). This miR2863c can target the promoter region of *OsNAC300*, and the target promoter region exhibits methylation differences between WT and *Osago2* mutants (psRNATarget; https://www.zhaolab.org/psRNATarget/) (Figure 3E, F). RNA immunoprecipitation results confirmed that, miR2863c was associated with OsAGO2. These results suggested that OsAGO2 bound to miR2863c, and the miR2863c/OsAGO2 RISC can suppress the *OsNAC30*0 transcription by mediating the DNA methylation of the *OsNAC30*0 promoter to maintain the normal senescent process of leaves.

### Overexpression of *OsNAC300* causes premature leaf senescence

**Figure 4:**
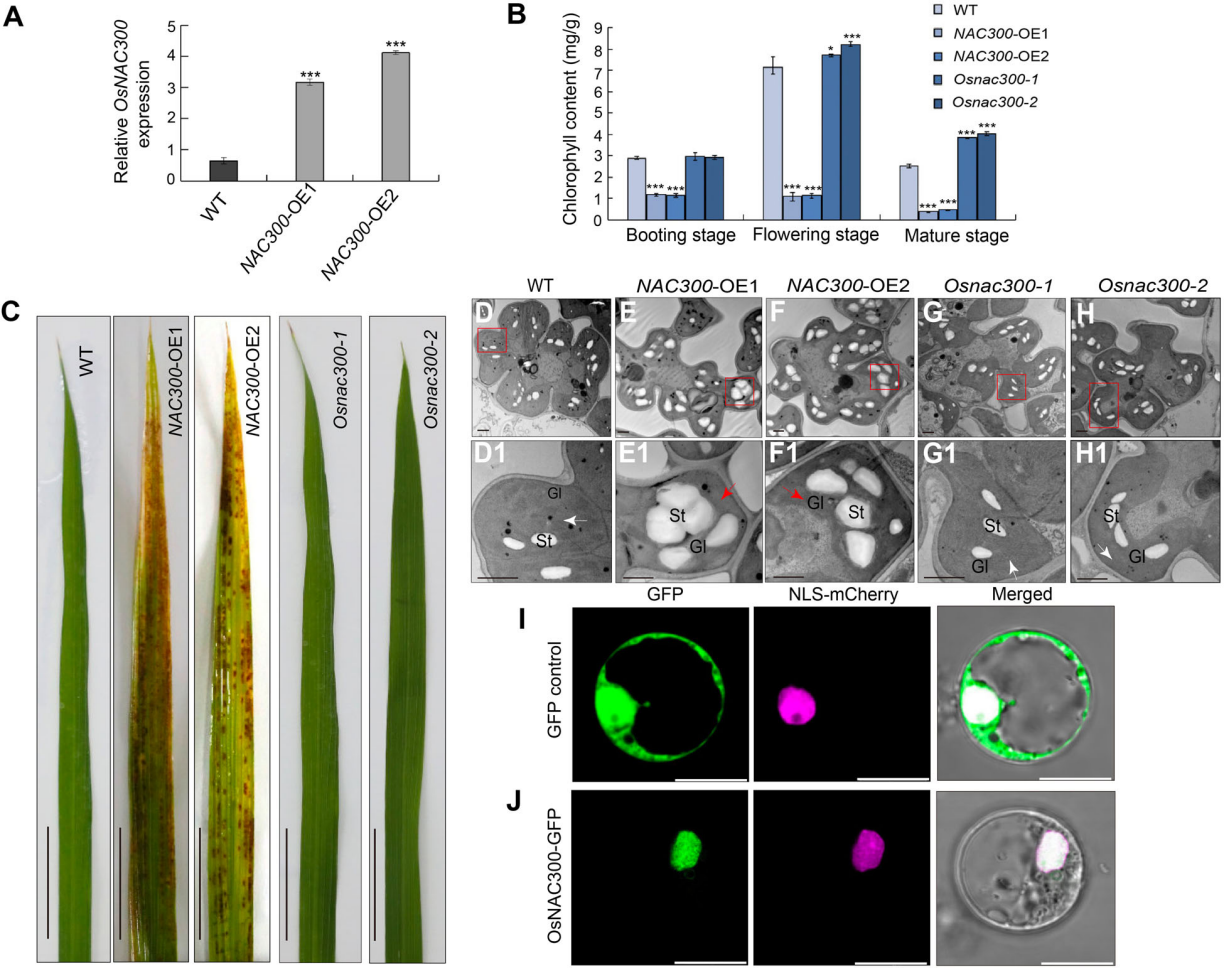
OsNAC300 mutants display early leaf senescence traits **(A)** Relative *OsNAC300* expression in leaves of wild‐type (WT), *NAC300*‐OE1, and *NAC300*‐OE2 plants at the flowering stage. All data are presented as means ± *SD* of three independent replicates. ***P < 0.001. *P*‐values were determined by Student's *t*‐test. **(B)** Chlorophyll contents of WT, *NAC300*‐OE1, *NAC300*‐OE2, *Osnac300‐1*, and *Osnac300‐2* plants from the booting to mature stages. All data are presented as means ± *SD* of three independent replicates. ***P < 0.001. *P*‐values were determined by Student's *t*‐test. **(C)** Representative photographs of flag leaves from WT, *NAC300*‐OE1, *NAC300*‐OE2, *Osnac300‐1*, and *Osnac300‐2* plants at the flowering stage. Bars = 3 cm. **(D** ***–*** **H1)** Transmission electron microscopy (TEM) analysis of leaves from WT, *NAC300*‐OE1, *NAC300*‐OE2, *Osnac300‐1*, and *Osnac300‐2* plants at the flowering stage. **(D1)** to **(H1)** are the enlarged images in red box from **(D)** to **(H)**. The arrows showed the abnormal (red) or normal (white) grana lamellae. GL, grana lamellae, St, starch grain. Bars = 1 µm. **(I, J)** Subcellular localization of green fluorescent protein (GFP) control (upper panels) and OsNAC300‐GFP (bottom panels) in transformed rice sheath protoplasts. The nuclear localization signal‐mCherry fusion protein served as a nuclear marker. Bars = 10 μm.

To determine whether changing the expression levels of *OsNAC300* influences leaf senescence, we generated *OsNAC300* OE lines and *Osnac300* mutants via gene editing, both in the ZH11 background. We chose two stable transgenic OE lines for phenotypic characterization, named *NAC300*‐OE1 and *NAC300*‐OE2, each showing three‐ to four‐times higher *OsNAC300* transcript levels relative to the WT (Figure 4A). The CRISPR/Cas 9 lines, named *Osnac300‐1* and *Osnac300‐2*, harbored premature stop codons (Figure S4A). From the booting stage to the mature stage, *NAC300*‐OE1 and *NAC300*‐OE2 showed premature leaf phenotypes and significantly lower total chlorophyll contents, increased hydrogen peroxide contents, and lower seed‐setting rates relative to the WT (Figures 4B, C, S4B, C). The *Osnac300‐1* and *Osnac300‐2* mutants had leaves with a normal green color, higher total chlorophyll contents than WT leaves, normal hydrogen peroxide contents, and normal seed‐setting rates (Figures 4B, C, S4B, C). We also examined the chloroplast ultrastructure of leaves from WT, *Osnac300‐1*, and *Osnac300‐2* plants, and found that their chloroplasts were filled with normal and dense thylakoid grana and several starch granules at the flowering stage (Figure 4D, D1, G, G1, H, H1). By contrast, the chloroplasts from *NAC300*‐OE1 and *NAC300*‐OE2 leaves had degraded thylakoid grana lamellae with abnormal starch granules (Figure 4E, E1, F, F1). These results indicate that OsNAC300 positively regulates leaf senescence.

Because the phenotypes of *NAC300*‐OE1 and *NAC300*‐OE2 plants were similar to those of *Osago2‐1* and *Osago2‐2* plants, and in light of the ChIP‐qPCR results showing that OsAGO2 binds to the *OsNAC300* promoter, we reasoned that *OsNAC300* expression is directly regulated by OsAGO2. To test this hypothesis genetically, we knocked out *OsNAC300* using CRISPR/Cas9 in the *Osago2‐1* background. We selected two lines for further analysis: *Osago2‐1/Osnac300‐1* and *Osago2‐1/Osnac300‐2* (Figure S5A). These plants showed normal leaf development with decreased *OsNAC300* and *OsAGO2* transcript levels compared with the WT (Figure S5A, B). The chloroplasts of *Osago2‐1/Osnac300‐1* and *Osago2‐1/Osnac300‐2* leaves presented a normal ultrastructure, which aligned well with the normal photosynthetic activity measured in these plants, based on F_v_/F_m_ values (Figure S5C–L) and partial recovery of seed‐setting rate and pollen fertility (Figure S5H, I).

These results suggest that OsAGO2 binds to the *OsNAC300* promoter and directly regulates its transcription via DNA methylation, thereby ensuring normal leaf function and proper onset of senescence.

### OsNAC300 regulates the expression of SAGs

NAC proteins constitute a major transcription factor family that is well known for its roles in plant growth, development, and responses to abiotic and biotic stresses. The NAM domain is a highly conserved N‐terminal domain with DNA binding function in NAC proteins. To explore the evolutionary relationships of OsNAC300 with other NAC proteins known to regulate leaf senescence (Cao et al., 2023), we performed a multiple sequence alignment using the entire protein sequences (including the NAM domain) of OsNAC300 and other NAC proteins in rice and Arabidopsis. Phylogenetic analysis revealed that OsNAC300 is highly similar to OsNAC2 (72.6%; Mao et al., 2017; Chun, et al., 2023) in rice and to ANAC092 (75.0%; Oh et al., 1997; Woo et al., 2004) and ANAC087 (74.4%; Podzimska‐Sroka et al., 2015) in Arabidopsis (Figure S6). This structural similarity suggests that they may all contribute to plant growth and leaf senescence. To investigate the subcellular localization of OsNAC300, we cloned the full‐length coding sequence of *OsNAC300* in‐frame and upstream of the sequence encoding green fluorescent protein (GFP) under the control of the cauliflower mosaic virus (CaMV) 35S promoter, yielding *35S:OsNAC300‐GFP*. We transfected this construct into leaf sheath protoplasts prepared from ZH11 and observed GFP fluorescence in the nucleus, which colocalized with the red fluorescence from the nuclear marker mCherry fused to a nuclear localization signal (NLS; Figure 4I, J). These results demonstrate that OsNAC300 is a nucleus‐localized protein.

**Figure 5:**
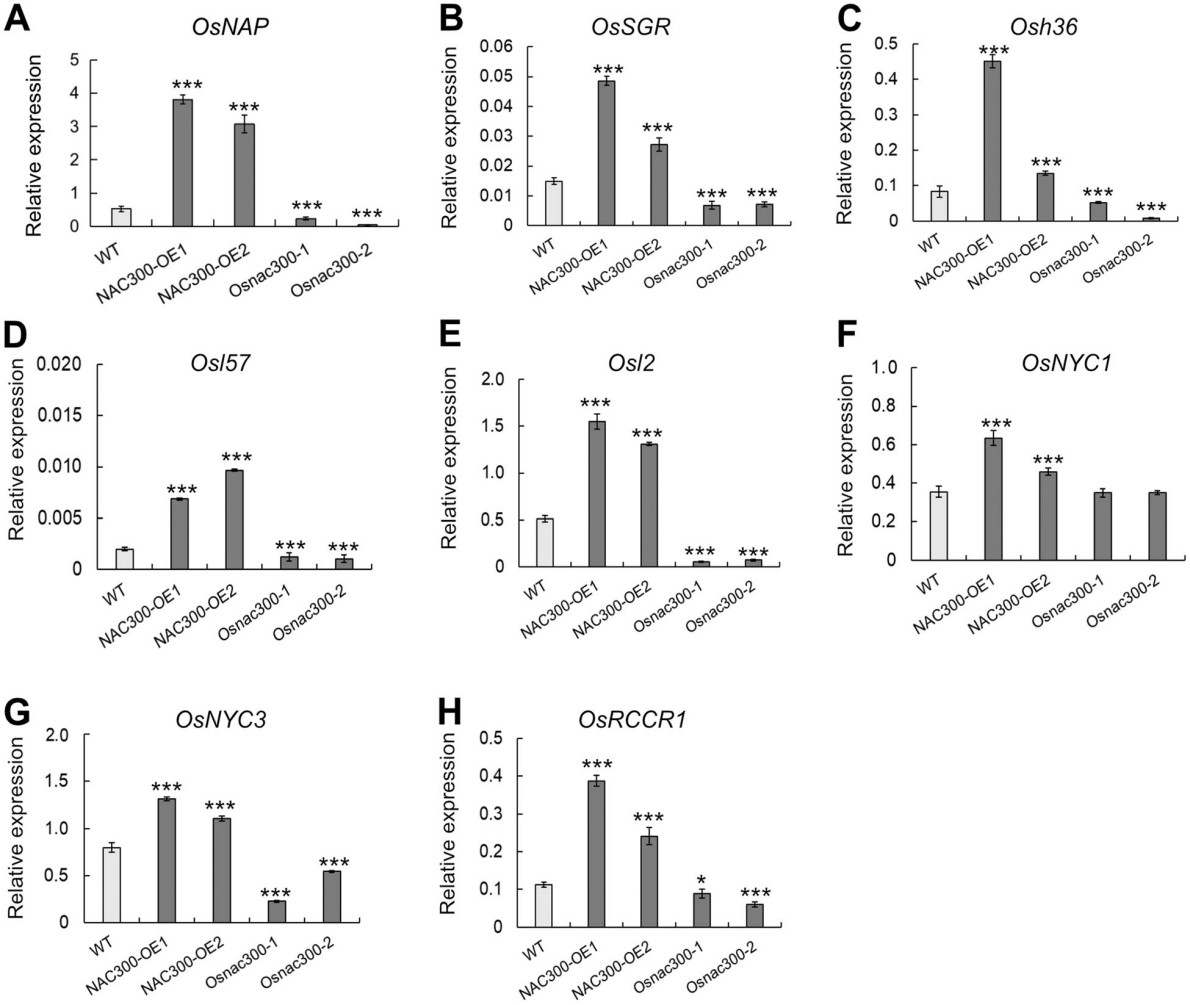
**OsNAC300 participates in leaf senescence by modulating** ***SAG*** **expression** **(A** ***–*** **H)** Relative expression of several *SAG* genes in wild‐type (WT), *NAC300*‐OE (overexpression), and *Osnac300* plants at the flowering stage. All data are presented as means ± *SD* from three independent replicates. *0.01 < *P* < 0.05, ***P < 0.001. *P*‐values were determined by Student's *t*‐test.

To identify downstream target genes of OsNAC300, we performed an RNA‐seq analysis of leaves collected from WT and *NAC300*‐OE plants at the flowering stage. We identified 3,832 DEGs in *NAC300*‐OEs relative to the WT, with 1,726 significantly upregulated genes (|log_2_ FC|≥ 1 and *Q*‐value < 0.05) and 2,106 downregulated genes (|log_2_ FC|≤ −1 and *Q*‐value < 0.05) (Figure S7A–C). We performed a Kyoto Encyclopedia of Genes and Genomes (KEGG) enrichment analyses to explore the biological functions associated with these upregulated DEGs and detected enrichment in several metabolic processes, such as “carbon metabolism” and “photosynthesis,” in *NAC300*‐OEs (Figure S7D, E). We also performed a Gene Ontology (GO) term enrichment analysis, revealing that these upregulated DEGs were mainly associated with “chloroplast,” “chloroplast stroma,” and “chloroplast thylakoid membrane” (Figure S7F, G). *SAG* genes also showed increased expression levels in *NAC300*‐OE plants compared with WT plants (Figure S7H). Given the low chlorophyll contents and abnormal chloroplast struction seen in *Osago2* mutants and *NAC300*‐OE lines, we further measured the expression levels of genes involved in chlorophyll degradation, chlorophyll biosynthesis, and chloroplast development in *NAC300*‐OE plants. Indeed, DEGs related to chlorophyll degradation were upregulated in *NAC300*‐OEs: *OsNAP* and *OsSGR*, serving as physiological senescence marker genes; *Osh36*, *Osl57*, and *Osl2*; *OsNYC1* and *OsNYC3*, encoding chlorophyll b reductase enzymes; and *OsRCCR1*, encoding red CHLOROPHYLL CATABOLITE REDUCTASE 1 (Figure 5A–H). These results suggest that OsNAC300 may directly or indirectly regulate the expression of several genes related to senescence.

### OsNAC300 mediates leaf senescence by regulating the expression of *OsNAP*

Since OsNAC300 is a typical NAC transcription factor located in the nucleus (Figure 4J), we performed DNA affinity purification followed by sequencing (DAP‐seq) to identify the DNA binding sites and the target genes of OsNAC300. In three biological repeats, the overlap in DAP‐seq data relative to the input (control) revealed 10,896 binding peaks associated with 6,151 genes distributed along all 12 chromosomes, with a high Pearson's correlation coefficient among replicates. Approximately 35% predicted binding sites mapped to promoter regions, with another third mapping to introns (Figure S8A). We conducted GO and KEGG enrichment analyses using the genes with OsNAC300 binding peaks in their promoter regions. These revealed that putative OsNAC300 targets are mainly involved in “key metabolic processes in plant development” and “DNA binding” (Figure S8B, C). Combining the results from DAP‐seq and RNA‐seq, we identified 109 genes bound by OsNAC300 and upregulated in *NAC300*‐OE lines (Figure S8D). These genes were mainly involved in “photosynthesis” and “response to ROS, response to biotic stimulus” (Figure S8E, F).

In the RNA‐seq dataset, we also noticed 16 upregulated NAC family genes in *NAC300*‐OE lines relative to the WT, most of which were associated with leaf senescence in previous research (Table S1). In addition, one of the marker genes for leaf senescence, *OsNAP* (Liang et al., 2014), was significantly upregulated in *NAC300*‐OE plants compared with WT plants (Figure 5A). RT‐qPCR result showed *OsNAC300* and *OsNAP* are mainly expressed in senescent leaves from the tillering stage to the senescent stage, and *OsNAP* was more highly expressed than *OsNAC300* in the senescent leaf (Figure S9A). However, *OsNAC300* was highly expressed in flag leaves of the *Osago2‐1* and *Osago2‐2* mutants, although they have not displayed clear signs of aging, and *OsNAP* showed lower expression levels at these same‐stage non‐senescent leaves in the *Osago2* mutants relative to the WT (Figure S9B). Thus, it suggests that *OsNAC300* is expressed earlier than *OsNAP* during leaf aging and is regulated by OsAGO2, suggesting that OsNAC300 may act upstream of *OsNAP*. A survey of the methylation levels of genes co‐upregulated in *Osago2* mutants relative to the WT and those co‐upregulated in *NAC300*‐OE plants compared with the WT revealed no significant difference in methylation levels between *Osago2* mutants and WT plants for these genes (Figure S3D; Table S4). Moreover, *OsNAP* expression was highly upregulated in *NAC300*‐OEs, suggesting that *OsNAP* may be regulated by OsNAC300.

**Figure 6:**
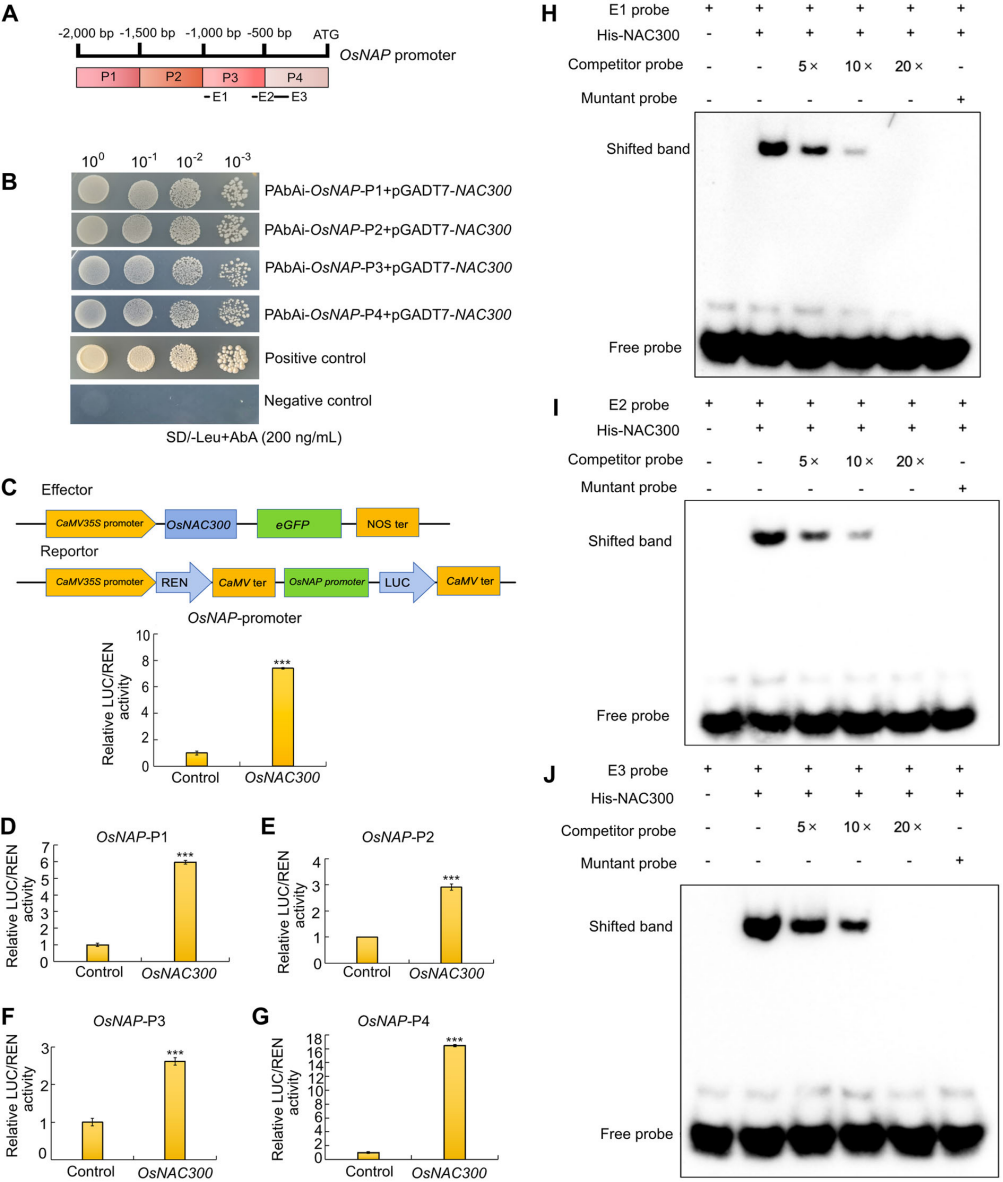
**OsNAC300 regulates leaf senescence by directly targeting the** ***OsNAP*** **promoter and promoting** ***OsNAP*** **transcription to induce early leaf senescence in rice** **(A, B)** Yeast one‐hybrid assay showing the interaction between OsNAC300 and different fragments (P1*–*P4) of the *OsNAP* promoter. P53‐AbAi was used as the positive control, and a negative control strain with a pMutant‐AbAi as the user manual protocol. Different dilutions of yeast cultures were spotted onto synthetic defined (SD) medium plates. **(C)** Diagrams of the reporter and effector vectors used for dual firefly luciferase (LUC) assays and the dual‐luciferase reporter assay was conducted for the OsNAC300 to the whole *OsNAP* promoter. All data are presented as means ± *SD* from five independent replicates. Asterisks indicate significant differences by Student's *t*‐test, ***P < 0.001. **(D** ***–*** **G)** OsNAC300 activates the transcription of *OsNAP* to activate leaf senescence. The LUC/REN (*Renilla*) ratio from the empty effector vector (*35S:eGFP*) plus promoter was set to 1. All data are presented as means ± *SD* from five independent replicates. Asterisks indicate significant differences by Student's *t*‐test, ***P < 0.001. **(H** ***–*** **J)** Electrophoretic mobility shift assays (EMSAs) showing the specific binding of OsNAC300 to three distinct binding regions in the *OsNAP* promoter *in vitro*. Unlabeled wild‐type (WT) probes (5‐, 10‐, and 20‐fold excess) were used as competitors. The mutant probe was used as the negative control. Probe positions are shown in **(A)** (E1*–*E3). Plus and minus symbols represent the presence and the absence of components, respectively.

To test this possibility, we examined the DAP‐seq results obtained for OsNAC300 and detected binding peaks for OsNAC300 in the *OsNAP* promoter region (Figure S8G). We determined whether OsNAC300 directly binds to the *OsNAP* promoter region by performing a Y1H assay, dual‐luciferase assay, and an EMSA (Figure 6A–J). A Y1H assay confirmed that OsNAC300 strongly binds to the *OsNAP* promoter over the four regions P1–P4 (Figure 6A, B). We generated reporter constructs consisting of whole and P1–P4 promoter regions of *OsNAP* driving transcription of the *LUC* reporter gene and used these constructs in a dual‐luciferase assay with a *35S:OsNAC300* construct as effector. Co‐transfection of rice protoplasts with each LUC reporter and the *35S*:*OsNAC300* effector construct resulted in higher relative LUC activity than transfection with the empty effector vector control (Figure 6C–G), indicating that OsNAC300 activates *OsNAP* transcription. We produced and purified recombinant HIS‐OsNAC300 from *Escherichia coli* cells for an EMSA. When incubated with DNA probes covering the E1–E3 binding sites in the *OsNAP* promoter, defined by DAP‐seq analysis, recombinant HIS‐OsNAC300 caused a shift in the mobility of each probe that was reversed under competition by excess unlabeled probes (Figure 6H–J). Plants overexpressing *OsNAP* (*ps1‐D*, a T‐DNA insertion mutant leading to a gain‐of‐function allele; Liang et al., 2014) were previously shown to exhibit premature leaf senescence phenotypes similar to those of *NAC300*‐OE plants. Notably, knocking out *OsNAP* in *NAC300*‐OE plants via CRISPR/Cas9 restored a WT leaf color, normal chloroplast struction, normal chlorophyll contents, and typical chlorophyll fluorescence parameters (F_v_/F_m_) (Figure S10).

### Overexpression of *OsAGO2* leads to delayed leaf senescence and increased grain yield

Furthermore, we also observed the delayed leaf senescence symptoms in the *OsAGO2*‐OE plants with more total chlorophyll contents than those of WT plants from the mature stage (Figure S11A, B, F). Expression analysis revealed the expression of *OsNAC300* was significant repressed with higher methylation level in the promoter regions in the *AGO2*‐OE plants (Figure S11C, D). We also found *AGO2*‐OE plants accumulated more total chlorophyll contents than those of WT plants which was consistent with the observed decline in *OsNAP* expression (Figure S11E, F). An examination of the agronomic traits related to grain yield in two *OsAGO2* OE plants, *AGO2*‐OE1 and *AGO2*‐OE2, showed a 4.0% and 4.7% increase in seed‐setting rate and a 5.4% and 3.3% increase in 100‐grain weight, a 35.6% and 31.6% increase in yield per plant, respectively, compared with WT (Figure S11G–I). Taken together, these results demonstrate that OsNAC300 directly binds to the *OsNAP* promoter and regulates its expression to play a crucial role in promoting early leaf senescence. *OsNAC300* expression itself is regulated by methylation of its promoter via OsAGO2. These results suggest that increasing the expression of *OsAGO2* can prolong the function of leaves and have the potential to increase yield.

## DISCUSSION

### Epigenetic regulation of leaf aging in rice by OsAGO2

Epigenetic modifications such as histone methylation, acetylation, and DNA cytosine methylation, regulate gene expression during plant growth and development as well as leaf senescence (Ay et al., 2014). However, whether and how DNA methylation contributes to aging in rice have not been studied. DNA methylation is an important mode of epigenetic regulation that mainly produces 5‐methylcytosine (5‐mC) in CG‐rich DNA segments and repetitive sequences such as the centromere region, transposon regions, and ribosomal RNA genes. DNA methylation regulates the expression of imprinted genes and maintains genome stability, with DNA methylation in promoter regions repressing transcription. In this study, we showed that the DNA methylation level along the *OsNAC300* promoter region was lower in leaves of *Osago2* mutants than in leaves of WT plants in the CG, CHG, and CHH contexts, as determined by bisulfite sequencing (Figures 3C, S3D). This finding suggests that the regulation of DNA methylation during plant leaf senescence is different from other aspects of plant biology that are under the control of DNA methylation.

ARGONAUTE proteins play a crucial role in sRNA‐mediated gene silencing. Studies in Arabidopsis have demonstrated that 24‐nt miRNAs directly affect DNA methylation and participate in epigenetic silencing during plant male gametophyte development. Among the AGO proteins of Arabidopsis, AGO4, AGO6, and AGO9 are mainly involved in RdDM (Qi et al., 2006; Tolia and Joshua‐Tor, 2007; Brabbs et al., 2013). In rice, OsAGO4 and OsAGO6 collaborate in DNA methylation at specific target sites (Wu et al., 2009). We previously revealed that OsAGO2 regulates PCD of tapetum cells in anthers through epigenetic DNA methylation, thus affecting pollen fertility (Zheng et al., 2019). In this study, we found that OsAGO2 regulates the expression of *OsNAC300* through DNA methylation and mediates the senescence process of rice leaves. Notably, the senescence phenotype of *Osago2* mutant leaves was restored by knocking out *OsNAC300*, but the fertility of anthers and the seed‐setting rate were not fully restored, suggesting that other downstream genes may be involved in regulating the development of these two traits (Figure S5H, I). The early leaf senescence phenotype of *Osago2‐1* and *Osago2‐2* plants suggested a function for *OsAGO2* in regulating rice leaf senescence. Importantly, we established here that OsAGO2 binds to the 24‐nt miR2863c, which reversely complements the region within the *OsNAC300* promoter (Figure 3). Our results suggest that miR2863c recruits OsAGO2 and other unidentified components to the *OsNAC300* promoter, which leads to DNA methylation and leaf senescence than for tapetum cell development.

As described in our previous research, OsAGO2 regulated the initiation of tapetal PCD in rice anthers (Zheng et al., 2019), we speculate that OsAGO2 regulates different target genes in leaves and anthers, and there are differences in the methylation patterns of the regulated genes. The methylation level of *OsNAC300* in WT senescent leaves is lower than that in normal non‐senescent leaves. We determined that the DNA methylation level along the *OsNAC300* promoter region is lower in the leaves of *Osago2* knockout plants than in those of WT plants. Compared with non‐senescent leaves, the methylation level of *OsNAC300* decreased more significantly in the early senescence leaves of the *ago2* mutant (Figure 3B). ChIP‐qPCR assays showed that OsAGO2 binds to the *OsNAC300* promoter region, thus directly connecting OsAGO2‐mediated regulation of DNA methylation to *OsNAC300* expression (Figure 3D). We established a genetic link between OsAGO2, OsNAC300, and leaf senescence by knocking out *OsNAC300* in the *Osago2* background, in which *OsNAC300* is expressed at much higher levels than in the WT. *Osnac300*/*Osago*2 plants exhibited phenotypes similar to the WT, placing *OsNAC300* downstream of OsAGO2 (Figure S5). Based on the GO and KEGG analyses of DEGs, we propose that binding of OsAGO2 to the *OsNAC300* promoter region may affect the maintenance of leaf function, initiation of leaf senescence, and the accumulation of ROS levels in leaves.

### OsNAC300 induces leaf senescence by directly modulating *OsNAP* expression

Leaf senescence involves the downregulation of photosynthesis, chloroplast degradation, the degradation of nucleic acids, proteins, and lipids, as well as nutrient recycling from leaves to sink tissues (Lim et al., 2007; Mao et al., 2017). The NAC family of transcription factors is associated with leaf senescence (Gregersen and Holm, 2007). In rice, several NAC‐type transcription factors are reported to be related to leaf senescence, including *NAC WITH TRANSMEMBRANE MOTIF 1‐LIKE4* (*NTL4*; Lee et al., 2012), *OsNAC5* (Sperotto et al., 2009), *OsNAC6* (Nakashima et al., 2007), *OsORE1* (Kim et al., 2014), *ONAC106* (Sakuraba et al., 2015), and *OsNAP* (Liang et al., 2014). Of these, *OsNAP* directly controls the expression of *SAGs*, such as *OsSGR*, *OsNYC1*, *OsNYC3*, *OsRCCR1*, and *Osl57*, and plays a role in chlorophyll degradation (Sato et al., 2009; Tang et al., 2011; Liang et al., 2014). Similarly, ONAC106 directly controls the expression of *OsSGR* and *OsNYC1* (Sakuraba et al., 2015). In this study, overexpression of *OsNAC300* promoted the expression of *OsNAP*, indicating that OsNAC300 might act in the same pathway as *OsNAP* to modulate chlorophyll degradation.

Reactive oxygen species, commonly considered harmful by‐products of various metabolic pathways, also act as signaling molecules in normal plant development and participate in the regulation of PCD (Apel and Hirt, 2004). The accumulation of ROS is a common signal during aging. For instance, in Arabidopsis, ANAC032 positively regulates leaf senescence through the accumulation of hydrogen peroxide (Mahmood et al., 2016). We established here that *OsNAC300*‐overexpressing plants accumulate more ROS in their leaves than WT plants, suggesting that *OsNAC300* likely regulates leaf senescence by affecting chloroplast struction through the modulation of ROS levels. Additionally, compared with WT plants, *OsNAC300*‐overexpressing plants displayed premature leaf senescence characterized by lower chlorophyll contents and decreased photosynthetic activity. OsNAC300 localizes to the nucleus, where it induces the expression of the senescence‐related gene *OsNAP*. In summary, OsNAC300 is a positive regulator of senescence in rice, promoting premature leaf senescence by activating the expression of *OsNAP* by directly binding to the *OsNAP* promoter, as determined by Y1H assays, dual‐luciferase assays, and EMSAs (Figure 6).

More than 50% of NAC transcription factors appear to be associated with senescence (Guo and Gan, 2006; Liang et al., 2014; Podzimska‐Sroka et al., 2015; Mao et al., 2017; Cao et al., 2023). Our RNA‐seq analysis of leaves from WT, *NAC300*‐OE, and *Osago2* mutant plants uncovered other NAC family genes upregulated in *Osago2* mutant and *NAC300*‐OE plants relative to the WT (Table S1). We conclude that OsAGO2 and OsNAC300 modulate a common set of genes involved in regulation of leaf senescence. The DNA methylation level of the *OsNAC300* promoter was lower in the leaves of *Osago2* mutants than in those of the WT, while that of other *OsNAC* promoters remained unchanged, indicating that OsAGO2 indirectly induces the expression of other *OsNAC* genes by regulating the expression of *OsNAC300*. Indeed, the senescence marker gene *OsNAP* was upregulated in the *Osago2* mutants, but its promoter region had comparable methylation levels in *Osago2* mutants and in the WT (Table S4). We performed DAP‐seq assays, Y1H assays, dual‐luciferase reporter assays, and EMSAs, which all confirmed that OsNAC300 strongly binds to the *OsNAP* promoter region (Figure 6). In agreement, *OsNAP* expression was higher in *NAC300*‐OE plants than in WT plants, indicating that *OsNAP* may be regulated by OsNAC300. Overexpression of *OsNAP* produces premature leaf senescence phenotypes similar to those seen in *NAC300*‐OE and *Osago2* plants (Figure S10). Together, these results suggest that OsAGO2 mediates the expression of *OsNAC300* through DNA methylation, with *OsNAP* being a downstream target of OsNAC300.

Through the observation of leaf ultrastructure, we discovered that chloroplast struction was abnormal during the flowering stage of *OsNAC300*‐overexpressing plants, with degradation of the stromal lamellae and aberrant thylakoids (Figure 4D–H1). This stage corresponds to the peak period of leaf function, and premature leaf senescence during this time is likely to have a significant influence on rice yield. This suggests that OsNAC300 differs from previously reported NAC transcription factors because, besides its involvement in senescence, it is also associated with maintenance of leaf function.

**Figure 7:**
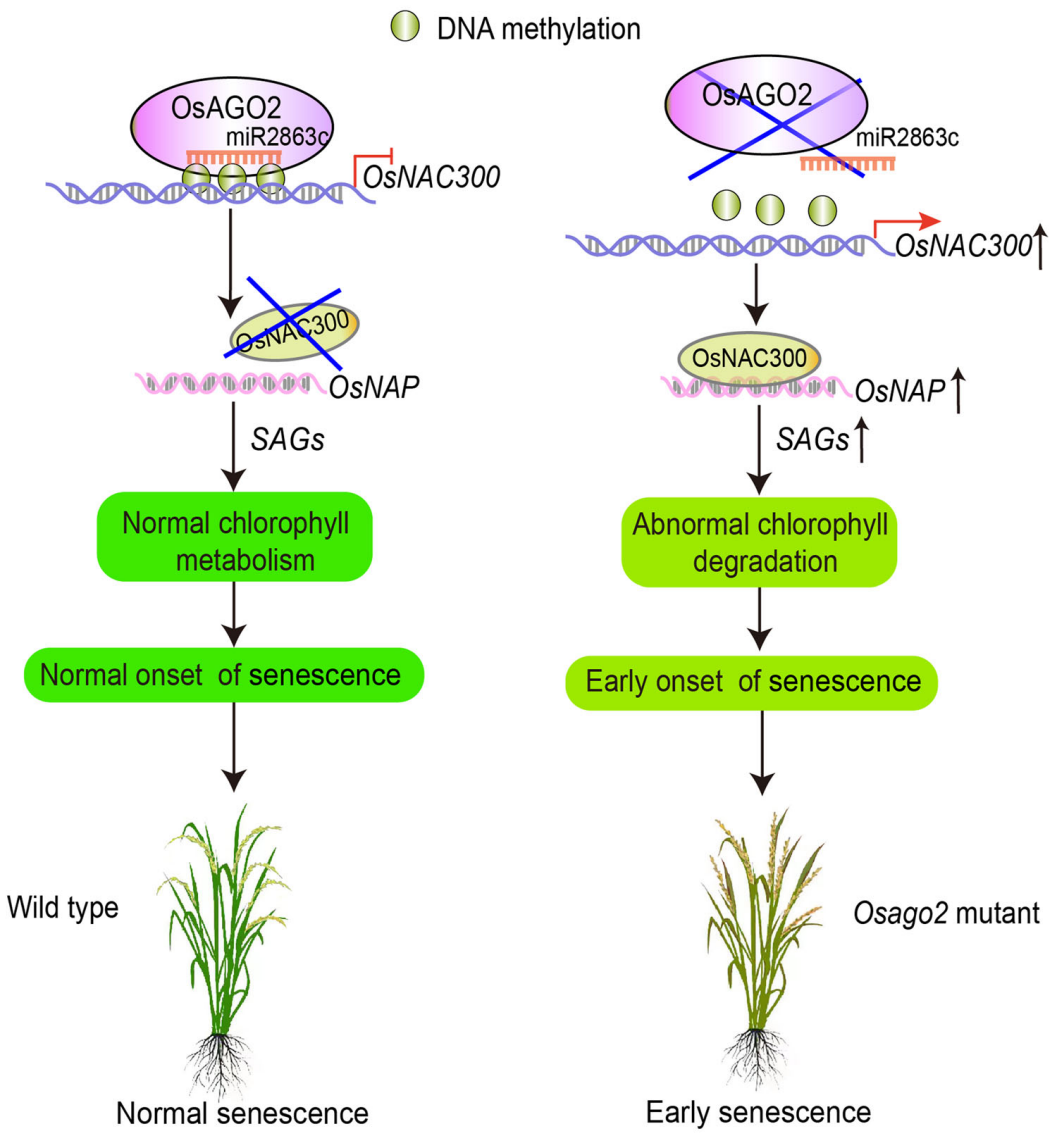
Proposed model for miR2863c/OsAGO2–*OsNAC300*–*OsNAP* in rice leaf senescence The flag leaves of *Osago2* mutant plants exhibit premature leaf senescence during the flowering stage. OsNAC300 is an important regulator of maintenance of leaf function in rice. OsAGO2 bound to miR2863c, and the miR2863c/OsAGO2 RISC controls *OsNAC300* transcription via modulating DNA methylation at its promoter, thereby ensuring proper initiation of aging. OsNAC300 participates in leaf senescence by modulating *OsNAP* expression, with accumulation of *OsNAC300* transcripts activating *OsNAP* expression. The leaf‐aging signal is triggered by the accumulation of *OsNAP* transcripts, and this accumulation initiates the normal onset of senescence. *OsNAP* then directly or indirectly regulates the expression of *SAG*s to control senescence in an age‐dependent manner. Thus, miR2863c/OsAGO2–*OsNAC300*–*OsNAP* is a key regulatory module linking the onset of senescence signaling and leaf senescence.

We propose a model for the OsAGO2 mediating epigenetic regulation module in rice leaf senescence (Figure 7). OsNAC300 is an important regulator of maintenance of leaf function in rice. OsAGO2 binds to the 24‐nt miR2863c, which reversely complements the promoter region within the *OsNAC300*. OsAGO2 controls *OsNAC300* expression by modulating DNA methylation of the *OsNAC300* promoter, thereby ensuring the proper initiation of leaf aging. A leaf‐aging signal is triggered by the accumulation of *OsNAP* transcripts, and this accumulation initiates the normal onset of senescence. OsNAP directly or indirectly regulates the expression of genes known to control senescence in an age‐dependent manner, including *SAG*s and chloroplast development‐related genes. In addition, OsNAC300 participates in leaf senescence by modulating *OsNAP* expression, as high *OsNAC300* expression levels lead to activation of *OsNAP* expression. Overexpression of *OsAGO2* not only delays leaf senescence but also improves the agronomic traits of rice, accompanied by repressing the expression of *OsNAC300* and *OsNAP*. Thus, OsAGO2–OsNAC300–*OsNAP* appears to act as a key regulatory module linking the onset of senescence signaling and leaf senescence.

## AUTHOR CONTRIBUTIONS

S.Z. and J.C. performed most of the research and S.Z., J.C., and Y.H. drafted the manuscript. Y.H. analyzed data and performed bioinformatics. J.Q.L. performed some protein expression and collected data. H.C., Z.L., J.Z., and J.L. participated in this study and performed some of the experiments. S.Z., Z. L., and C.Z. revised the manuscript. S.Z. and C.Z. designed the experiments, supervised the study. All authors read and approved of its content.

## CONFLICTS OF INTEREST

The authors declare no conflict of interest.

## Supporting information

Additional Supporting Information may be found online in the supporting information tab for this article: http://onlinelibrary.wiley.com/doi/10.1111/jipb.13766/suppinfo

**Figure S1.** Consequences of *OsAGO2* loss of function on leaf senescence development in Zhonghua 11
**Figure S2.** The analysis of the differentially expressed genes (DEGs) with *Osago2* plants compared to wild‐type (WT) (Zhonghua 11) leaves
**Figure S3.** The analysis of the differentially methylated regions (DMRs) with *Osago2* plants compared to wild‐type (WT) (Zhonghua 11) leaves by whole‐genome bisulfite sequencing (WGBS)
**Figure S4.** The traits of wild‐type and *OsNAC300* transgenic lines
**Figure S5.** The characteristic analysis of knockout plants of *OsNAC300* in *Osago2‐1* plants
**Figure S6.** Phylogenetic analysis of OsNAC300 and other known NAC proteins functioning in the leaf senescence
**Figure S7.** The analysis of the differentially expressed genes (DEGs) with *NAC300*‐OEs (overexpressions) compared to wild‐type (WT) (Zhonghua 11) leaves
**Figure S8.** The Gene Ontology (GO) and Kyoto Encyclopedia of Genes and Genomes (KEGG) analyses of genome‐wide distribution of OsNAC300 binding sites
**Figure S9.** The relative expression levels of *OsNAC300* and *OsNAP* in the wild‐type (WT) and *Osago2* mutants
**Figure S10.** The leaf characteristic analysis of knockout of *OsNAP* in *NAC300*‐OE (overexpression) plants
**Figure S11.** The leaf characteristic analysis and agronomic traits of *AGO2*‐OE plants
**Table S1.** The upregulated NAC family genes in the transcriptome data of *Osago2‐1* _wild‐type (WT) and *NAC300*‐OEs (overexpressions) versus wild‐type (WT) (|log2 fold change|≥ 1 and *Q*‐value < 0.05)
**Table S2.** Identified microRNAs associated with OsAGO2 by RNA immunoprecipitation—small RNA (RIP‐sRNA) sequencing with an anti‐Flag antibody
**Table S3.** The accession numbers used in the phylogenetic analysis
**Table S5.** Primers used in this study

**Table S4.** The genome‐wide methylation levels of co‐upregulated expressing genes in the transcriptome data of *Osago2* mutants versus wild‐type (WT) and *NAC300*‐OEs (overexpressions) versus WT

## Data Availability

All data are provided in the figures, tables, and supplementary information included in this manuscript. The sequence data used in this study can be found in the Rice Annotation Project (https://rapdb.dna.affrc.go.jp/), the RNA‐seq, WGBS data, RIP‐sRNA sequencing data, DAP‐seq datasets described in this article have been deposited into the Sequence Read Archive (SRA) (accession number, PRJNA1058681; https://www.ncbi.nlm.nih.gov/bioproject/PRJNA1058681; PRJNA1064473; https://www.ncbi.nlm.nih.gov/bioproject/PRJNA1064473).
