# Supplementary material for: The OsAGO2–OsNAC300–OsNAP module regulates leaf senescence in rice
Source: J Integr Plant Biol. 2024 Aug 22;66(11):2395–411. doi: 10.1111/jipb.13766 (PMC11583845; doi:10.1111/jipb.13766)
Supplement: Supplementary file 1 — Figure S1. Consequences of OsAGO2 loss of function on leaf senescence development in Zhonghua 11 Figure S2. The analysis of the differentially expressed genes (DEGs) with Osago2 plants compared to wild‐type (WT) (Zhonghua 11) leaves Figure S3. The analysis of the differentially methylated regions (DMRs) with Osago2 plants compared to wild‐type (WT) (Zhonghua 11) leaves by whole‐genome bisulfite sequencing (WGBS) Figure S4. The traits of wild‐type and OsNAC300 transgenic lines Figure S5. The characteristic analysis of knockout plants of OsNAC300 in Osago2‐1 plants Figure S6. Phylogenetic analysis of OsNAC300 and other known NAC proteins functioning in the leaf senescence Figure S7. The analysis of the differentially expressed genes (DEGs) with NAC300‐OEs (overexpressions) compared to wild‐type (WT) (Zhonghua 11) leaves Figure S8. The Gene Ontology (GO) and Kyoto Encyclopedia of Genes and Genomes (KEGG) analyses of genome‐wide distribution of OsNAC300 binding sites Figure S9. The relative expression levels of OsNAC300 and OsNAP in the wild‐type (WT) and Osago2 mutants Figure S10. The leaf characteristic analysis of knockout of OsNAP in NAC300‐OE (overexpression) plants Figure S11. The leaf characteristic analysis and agronomic traits of AGO2‐OE plants Table S1. The upregulated NAC family genes in the transcriptome data of Osago2‐1 _wild‐type (WT) and NAC300‐OEs (overexpressions) versus wild‐type (WT) (|log2 fold change|≥ 1 and Q‐value < 0.05) Table S2. Identified microRNAs associated with OsAGO2 by RNA immunoprecipitation—small RNA (RIP‐sRNA) sequencing with an anti‐Flag antibody Table S3. The accession numbers used in the phylogenetic analysis Table S5. Primers used in this study [file JIPB-66-2395-s002.docx]

**SUPPORTING INFORMATION**

**
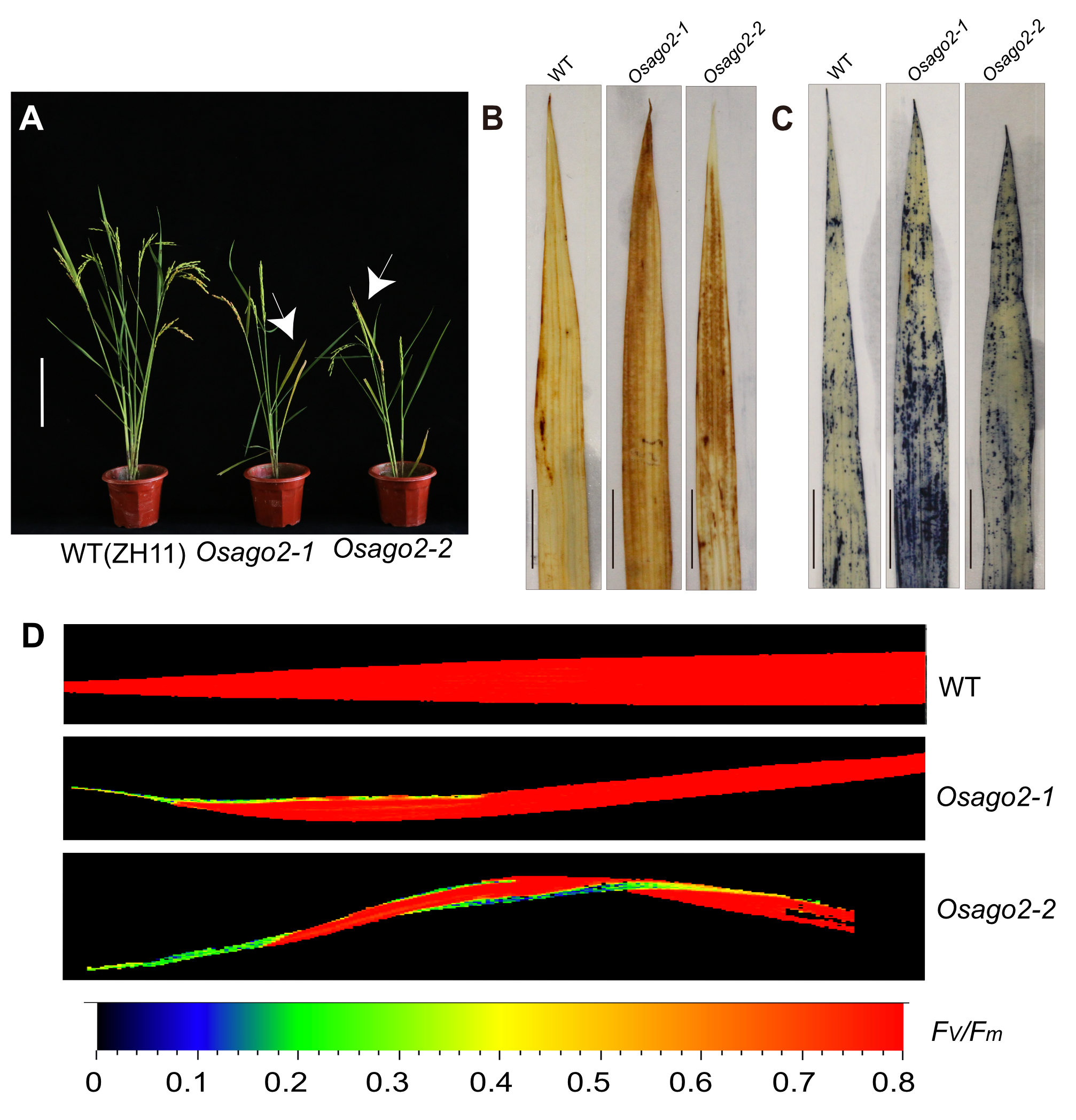
**

**Figure S1. Consequences of *OsAGO2* loss of function on leaf senescence development in Zhonghua 11.**

(A), Representative photographs of two independent transgenic plants of *OsAGO2*-CRISPR/Cas9 mutant lines of Zhonghua 11 (ZH11) grown in the greenhouse. All genotypes were transferred to pots at the flowering stage before taking the photographs. Bars = 20 cm.

(B), Representative DAB staining pattern of flag leaves from WT, *Osago2-1*, and *Osago2-2* plants at the flowering stage. Bars = 3 cm.

(C), Representative NBT staining pattern of flag leaves from WT, *Osago2-1*, and *Osago2-2* plants at the flowering stage. Bars = 3 cm.

(D), The chlorophyll fluorescence imaging analysis of WT (ZH11), *Osago2-1* and *Osago2-2* plants after the flowering stage.


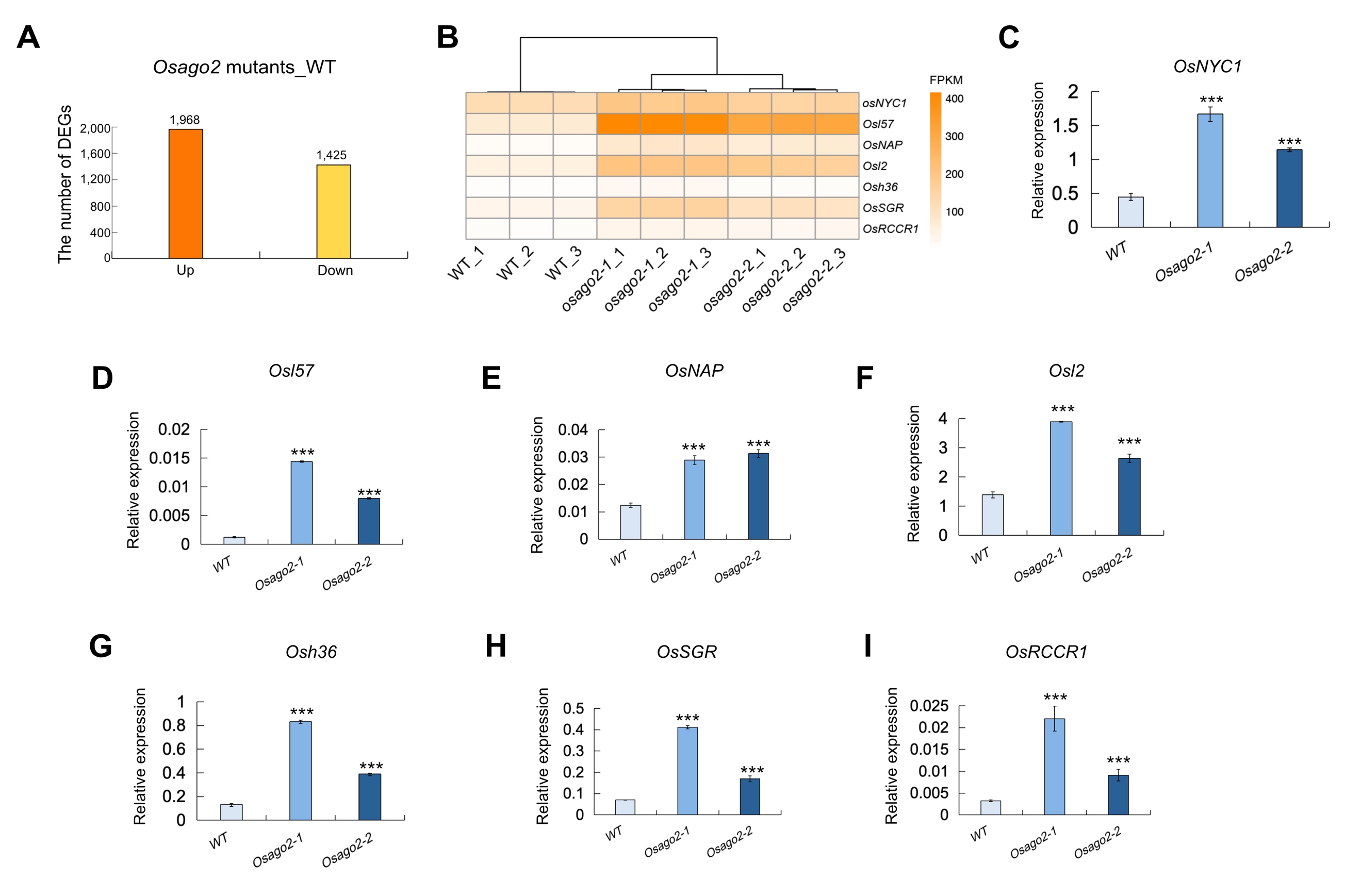


**Figure S2. The analysis of the differentially expressed genes (DEGs) with *Osago2* plants compared to wild-type (WT) (Zhonghua 11) leaves.**

(A), The numbers of up-regulated (|log2 FC| ≥ 1 and Q value < 0.05) and down-regulated (|log2 FC| ≤-1 and Q value < 0.05) DEGs in *Osago2* mutants compared to WT (Zhonghua 11) leaves.

(B), The heatmap of senescence-associated genes (SAGs) function in the leaf senescence in *Osago2* mutants compared to WT (Zhonghua 11) leaves.

(C-I), Relative expression of several *SAG* genes in WT, *Osago2-1* and *Osago2-2* plants at the flowering stage. All data are presented as means ± SD from three independent replicates. ***, *P* < 0.001. *P*-values were determined by Student’s *t-*test.

**
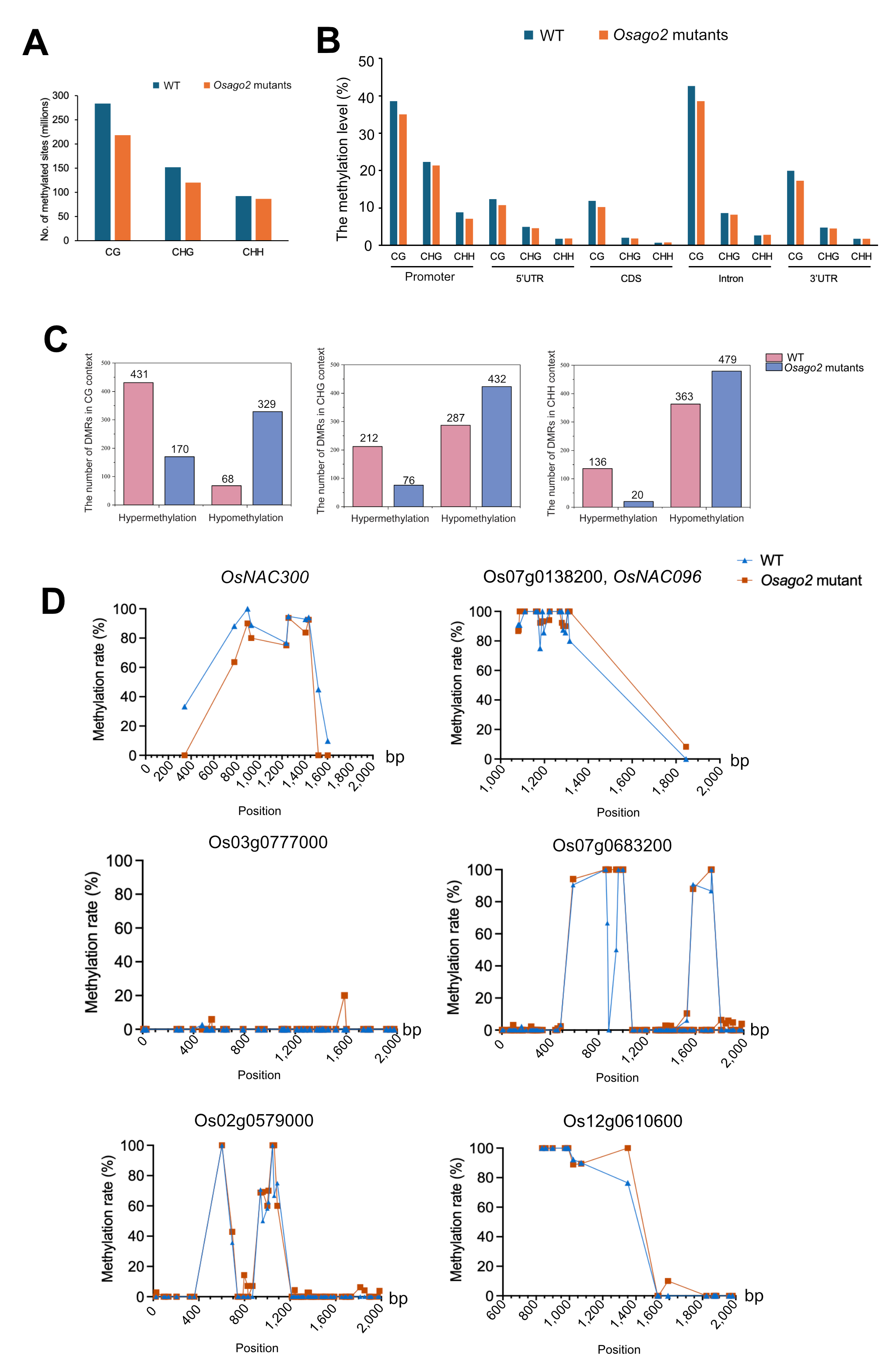
**

**Figure S3. The analysis of the differentially methylated regions (DMRs) with *Osago2 plants* compared to wild-type (WT) (Zhonghua 11) leaves by whole-genome bisulfite sequencing (WGBS).**

(A-B), Differential methylation patterns of WT and *Osago2* mutants. Methylation levels were quantified at CG, CHG, and CHH nucleotide contexts across gene bodies, intergenic regions, and 5’/3’ UTR regions within the rice genome.

(C), Details of DMRs in WT and *Osago2* during leaf aging (|log2 FC|≥ 1 and P value < 0.05). Hypermethylation, methylation levels ≥ 50%, Hypomethylation, methylation levels < 50%.

(D), The methylation levels of the upregulated NAC family genes in the transcriptome data of *Osago2* vs WT (|log2 FC|≥ 1 and P value < 0.05) detected by whole-genome bisulfite sequencing (WGBS).


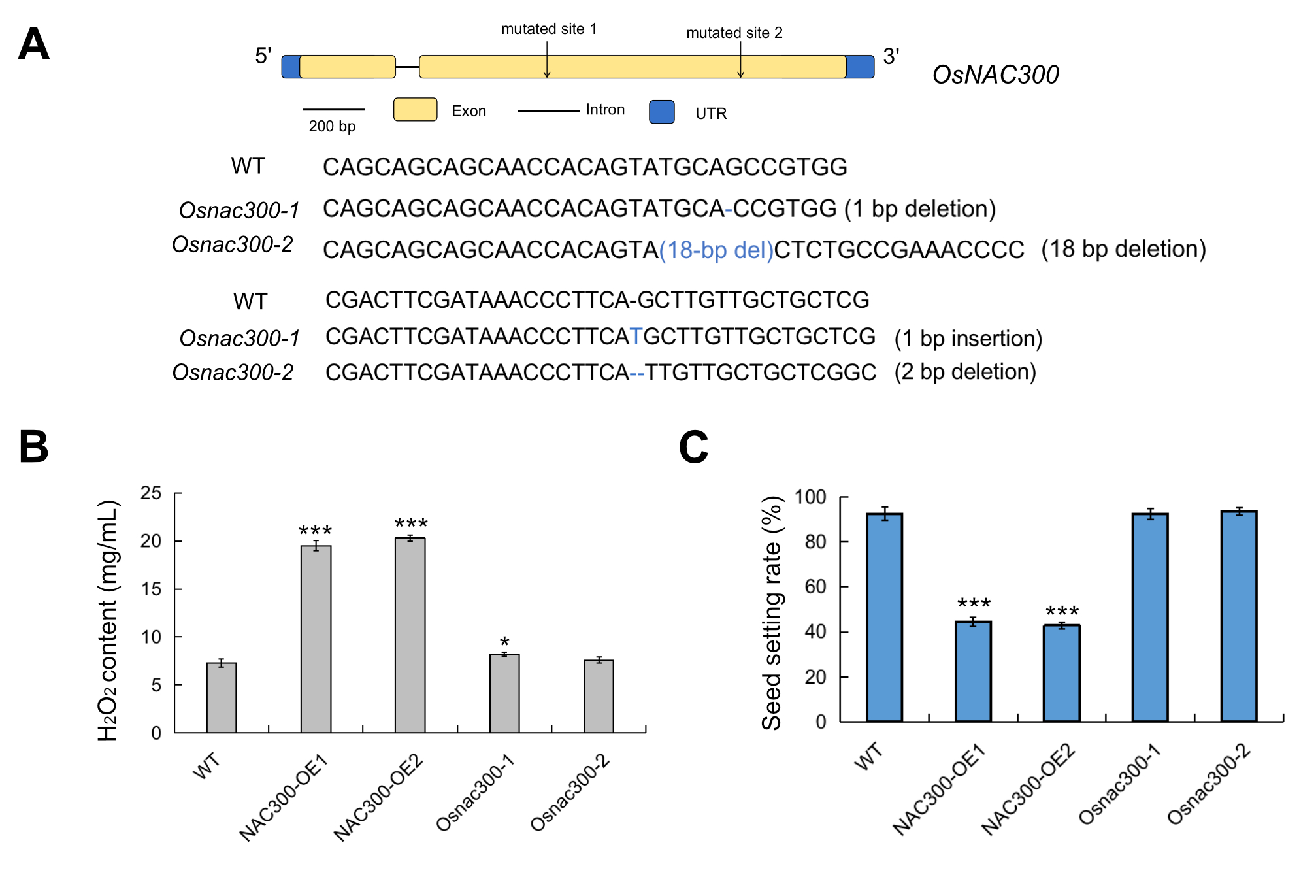


**Figure S4. The traits of wild-type and *OsNAC300* transgenic lines.**

(A), Mutated sites in the *Osnac300-1, Osnac300-2* mutant plants. The wild-type (WT) sequence is shown above, and the target sequence is highlighted in blue. Deleted bases are shown as “–”.

(B), The H_2_O_2_ contents in leaves of WT, *NAC300*-OEs, and *Osnac300* mutant plants at the flowering stage. *, 0.01 < P < 0.05, ***, P < 0.001. The P value was determined by Student’s *t-*test. All data are presented as means ± SD of three independent replicates.

(C), The seed setting rates of WT, *NAC300*-OEs, and *Osnac300* mutant plants. ***, P < 0.001. The P value was determined by Student’s *t-*test. All data are presented as means ± SD of three independent replicates.

**
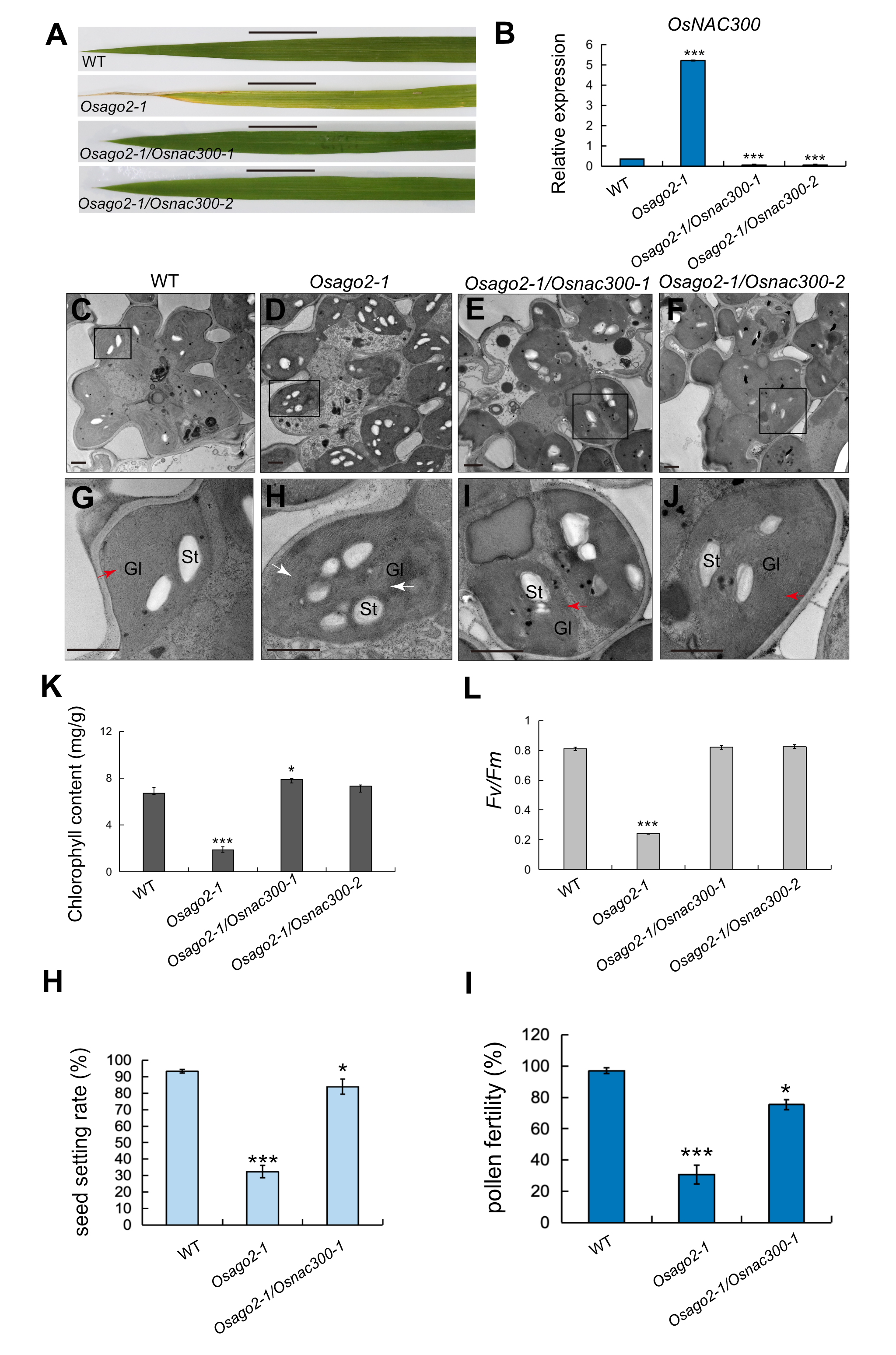
**

**Figure S5. The characteristic analysis of knockout plants of *OsNAC300* in *Osago2-1* plants.**

(A), The comparison of the flag leaf phenotype of WT, *Osago2-1*, and *Osago2-1/Osnac300-1, Osago2-1/Osnac300-2* mutant plants at the flowering stage. Bars = 3 cm.

(B), Relative expression of *OsNAC300* of WT, *Osago2-1*, and *Osago2-1/Osnac300-1, Osago2-1/Osnac300-2* leaves at the flowering stage. All data are presented as means ± SD of three independent replicates. ***, P < 0.001. P values were determined by Student’s *t-*test.

(C*–*J), TEM analysis of leaves of WT, *Osago2-1*, and *Osago2-1/Osnac300-1, Osago2-1/Osnac300-2* mutant plants at the flowering stage. GL, grana lamellae, St, starch grain. (G*–*J) are the enlarged images in black box from (C*–*F). Bars = 1 µm.

(K), Chlorophyll content analysis of WT, *Osago2-1*, and *Osago2-1/Osnac300-1, Osago2-1/Osnac300-2* plants at the flowering stage. All data are presented as means ± SD of three independent replicates. *, 0.01 < P < 0.05. ***, P < 0.001. P values were determined by Student’s *t-*test.

(L), *Fv*/*Fm* ratio analysis of WT, *Osago2-1*, and *Osago2-1/Osnac300-1, Osago2-1/Osnac300-2* mutant plants at the flowering stage. All data are presented as means ± SD of three independent replicates. ***, P < 0.001. P values were determined by Student’s *t-*test.

(H-I), Comparison of the seed setting rates and pollen fertility of WT, *Osago2-1*, and *Osago2-1/Osnac300-1* plants. Data are shown as means ± SD (n = 10 plants); asterisks indicate significant differences (*, 0.01<P < 0.05; ***, P < 0.001) according to Student’s *t*-test.


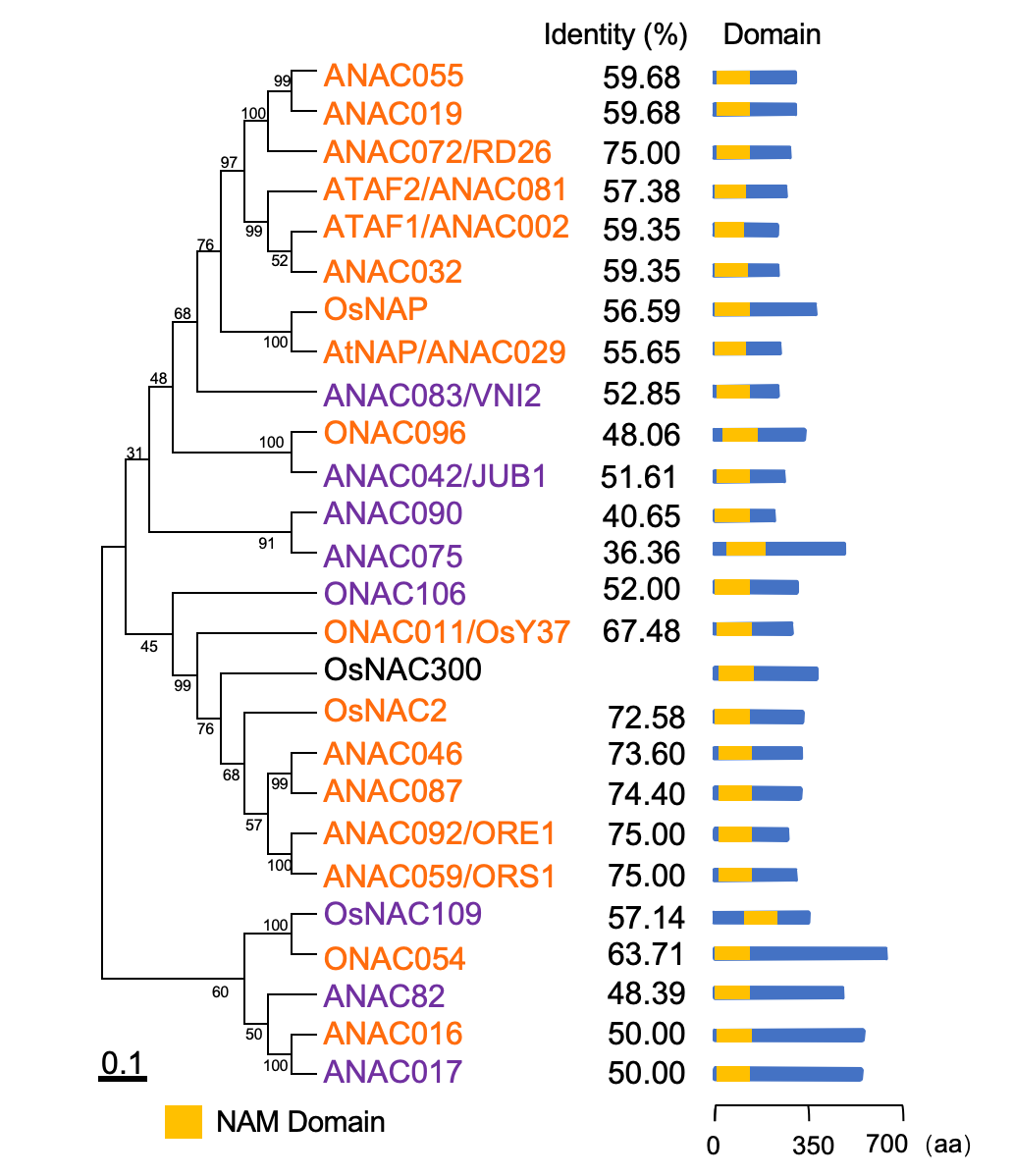


**Figure S6. Phylogenetic analysis of OsNAC300 and other known NAC proteins functioning in the leaf senescence.**

The protein sequences from rice and *Arabidopsis thaliana* were aligned with ClustalW-Align in MEGA 11 before reconstructing a maximum likelihood tree using the Construct Maximum Likelihood Tree method. The left side shows the phylogenetic tree. The right side indicates the protein domains identified by CCD program, and different domains are indicated by different colors at the bottom. The percentages given in the middle indicates the similarity of each protein to OsNAC300. OsNAC300 is in black. The orange font represents the proteins positively regulating leaf senescence, and the purple font represents the proteins negatively regulating leaf senescence. Tree bar = 0.1. The accession numbers for each protein are listed in Supplemental Table S3.


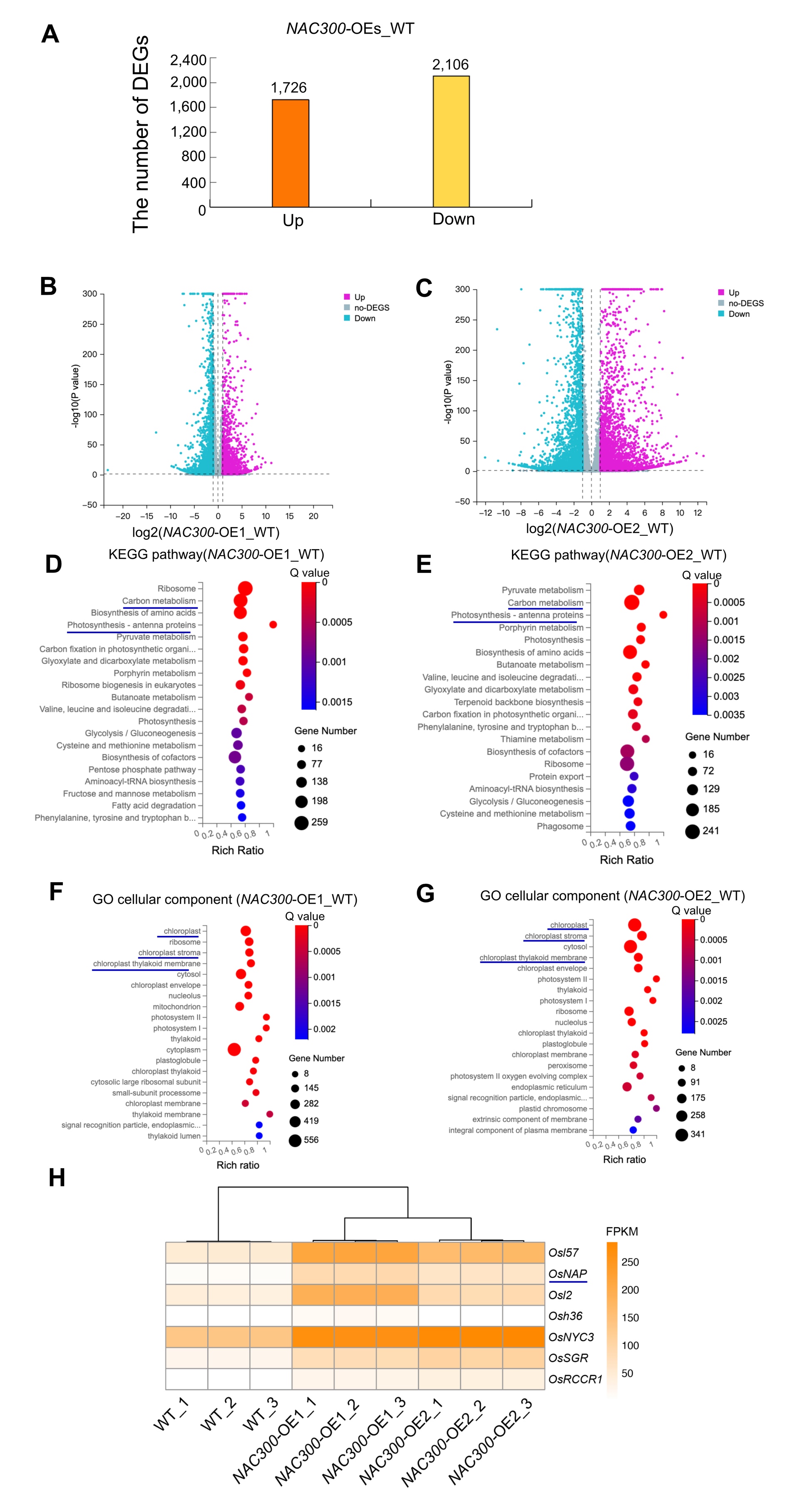


**Figure S7. The analysis of the differentially expressed genes (DEGs) with *NAC300*-OEs (overexpressions) compared to wild-type (WT) (Zhonghua 11) leaves.**

(A-C), The up-regulated (|log2 FC| ≥ 1, and Q value < 0.05) and down- regulated DEGs (|log2 FC| ≤ -1 and Q value < 0.05) in *NAC300*-OEs compared to WT (Zhonghua 11) leaves.

(D-G), The KEGG and GO analyses of genes with up-regulated DEGs (|log2 FC| ≥ 1 and Q value < 0.05) in *NAC300*-OEs compared to WT (Zhonghua 11) leaves.

(H), The heatmap of senescence-associated genes (SAGs) function in the leaf senescence in *NAC300*-OEs compared to WT (Zhonghua 11) leaves.


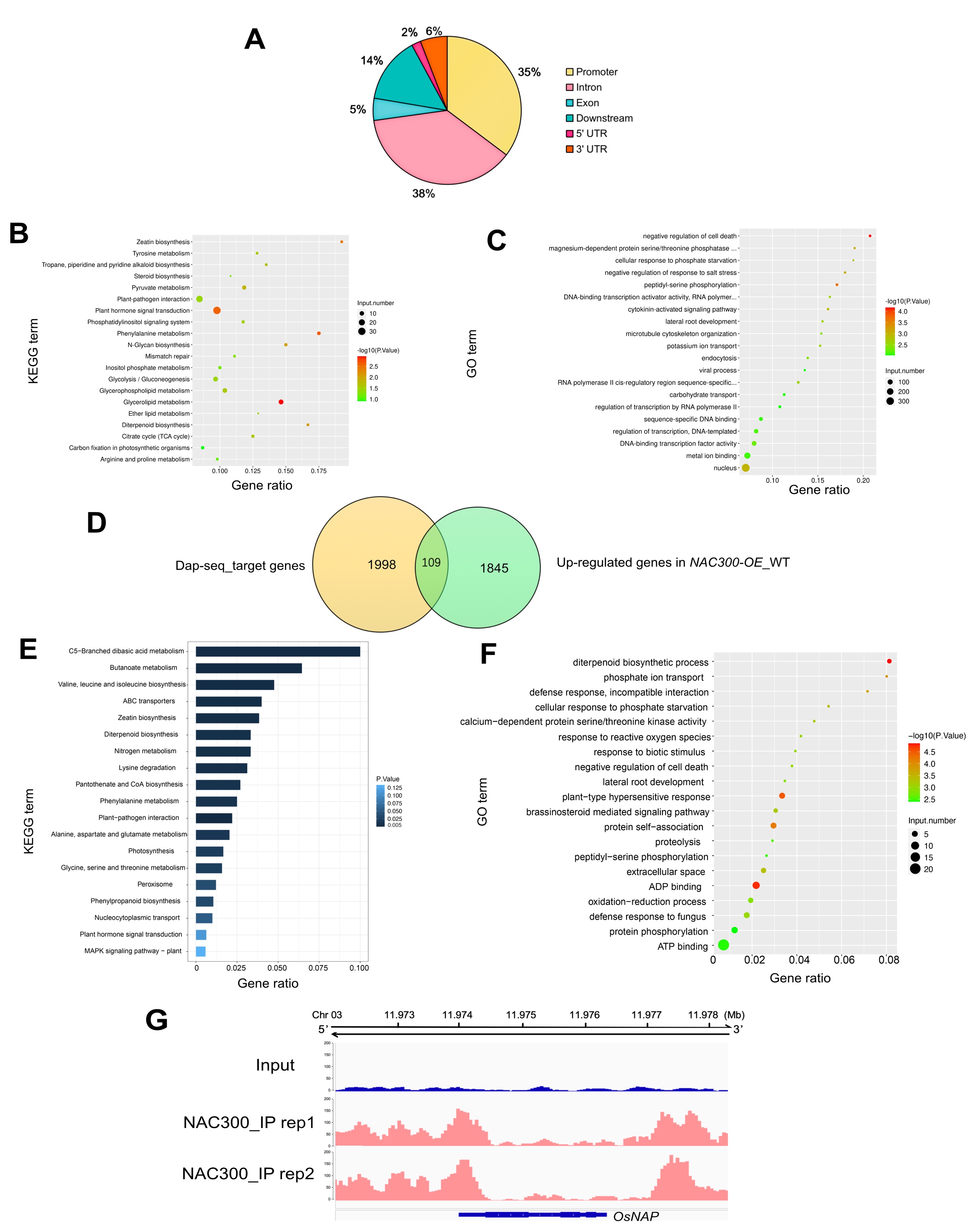


**Figure S8. The Gene Ontology (GO) and Kyoto Encyclopedia of Genes and Genomes (KEGG) analyses of genome-wide distribution of OsNAC300 binding sites.**

1. , Distribution of NAC300-binding sites in different regions of annotated genes by DNA affinity purification sequencing (DAP-seq).

(B, C), KEGG (Kyoto Encyclopedia of Genes and Genomes pathway classification) (B) and GO (Gene Ontology categorization) (C) analyses of NAC300-binding genes.

(D), Venn diagram showing the overlap between the genes bound by OsNAC300 and differentially up-regulated genes in RNA-seq dataset of *OsNAC300*-OE compared to WT (Zhonghua 11) leaves.

(E, F), The KEGG and GO analyses of 109 overlap genes in (D).

(G), Screenshots of OsNAC300 binding peaks in the promoter of *OsNAP* detected by DAP-seq (DNA affinity purification sequencing).

**
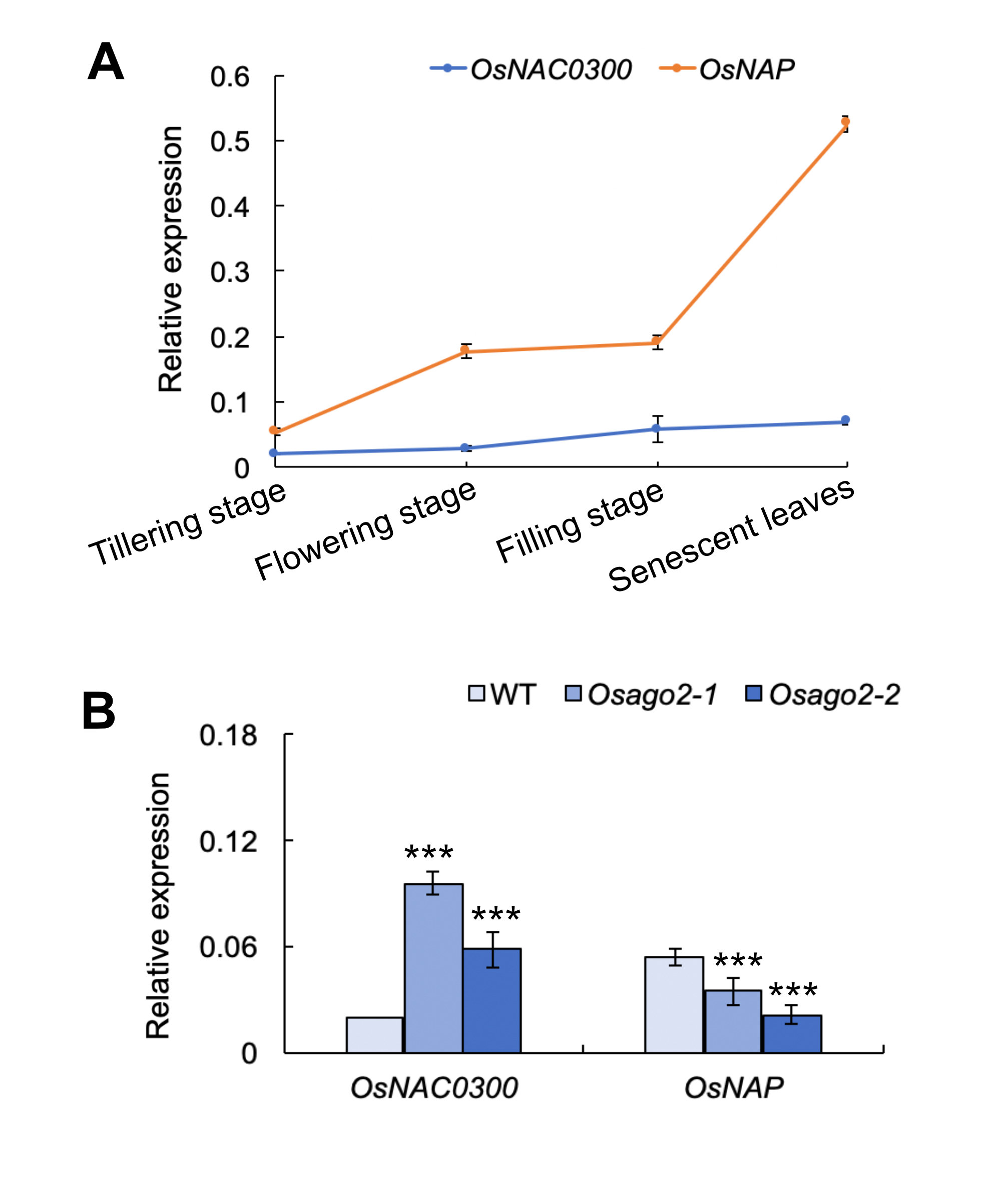
**

**Figure S9. The relative expression levels of *OsNAC300* and *OsNAP* in the wild-type (WT) and *Osago2* mutants.**

(A), Relative expression levels of *OsNAC300* and *OsNAP* at different leaf stages in WT. All data are presented as means ± SD of three independent replicates.

(B), Relative expression levels of *OsNAC300* and *OsNAP* at the fully expanded non-senescent leaves with no visible sign of yellowing of the WT, *Osago2-1* and *Osago2-2* plants. All data are presented as means ± SD of three independent replicates. ***, P < 0.001. P values were determined by Student’s *t-*test.

**
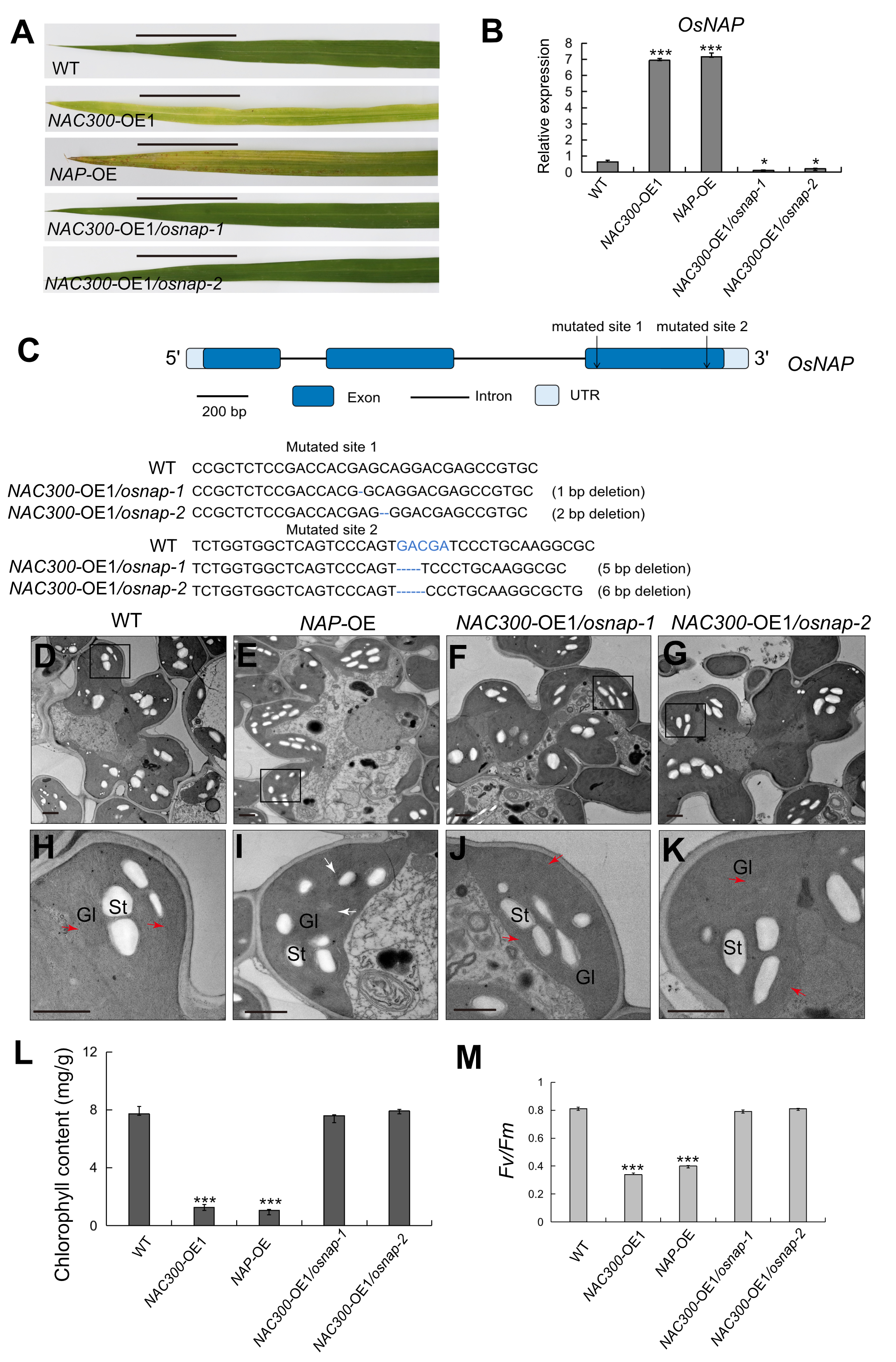
**

**Figure S10. The leaf characteristic analysis of knockout of *OsNAP* in *NAC300*-OE (overexpression) plants.**

(A), The comparison of the flag leaf phenotype of WT, *NAP-*OE (*ps1-D*, Liang et al., 2014), *NAC300-*OE1*/osnap-1, NAC300-*OE1*/osnap-2* plants at the flowering stage. Bars = 3 cm.

(B), Relative expression of *OsNAP* of WT, *NAP-*OE (*ps1-D*, Liang et al., 2014), *NAC300-*OE1*/osnap-1, NAC300-*OE1*/osnap-2* leaves at the flowering stage. All data are presented as means ± SD of three independent replicates. *, 0.01 < P < 0.05. ***, P < 0.001. P values were determined by Student’s *t-*test.

(C), Mutated sites and sequences of *OsNAP* in the WT, *NAC300-*OE1*/osnap-1, NAC300-*OE1*/osnap-2* plants. The wild-type sequence is shown above, and the target sequence is highlighted in blue. Deleted bases are shown as –.

(D*–*K), TEM analysis of leaves of WT, *NAP-*OE (*ps1-D*, Liang et., 2014), *NAC300-*OE1*/osnap-1, NAC300-*OE1*/**osnap-2* plants at the flowering stage. The arrows showed the abnormal (white) and normal (red) grana lamellae. GL, grana lamellae, St, starch grain. (H*–*K) are the enlarged images in black box from (D*–*G). Bars = 1 µm.

(L), Chlorophyll content analysis of WT, *NAP-*OE (*ps1-D*, Liang et al., 2014), *NAC300-*OE1*/osnap-1, NAC300-*OE1*/osnap-2* plants at the flowering stage. All data are presented as means ± SD of three independent replicates. ***, P < 0.001. P values were determined by Student’s *t-*test.

(M), Fv/Fm ratio analysis of WT, *NAP-*OE (*ps1-D*, Liang et al., 2014), *NAC300-*OE1*/osnap-1, NAC300-*OE1*/osnap-2* plants at the flowering stage. All data are presented as means ± SD of three independent replicates. ***, P < 0.001. P values were determined by Student’s *t-*test.


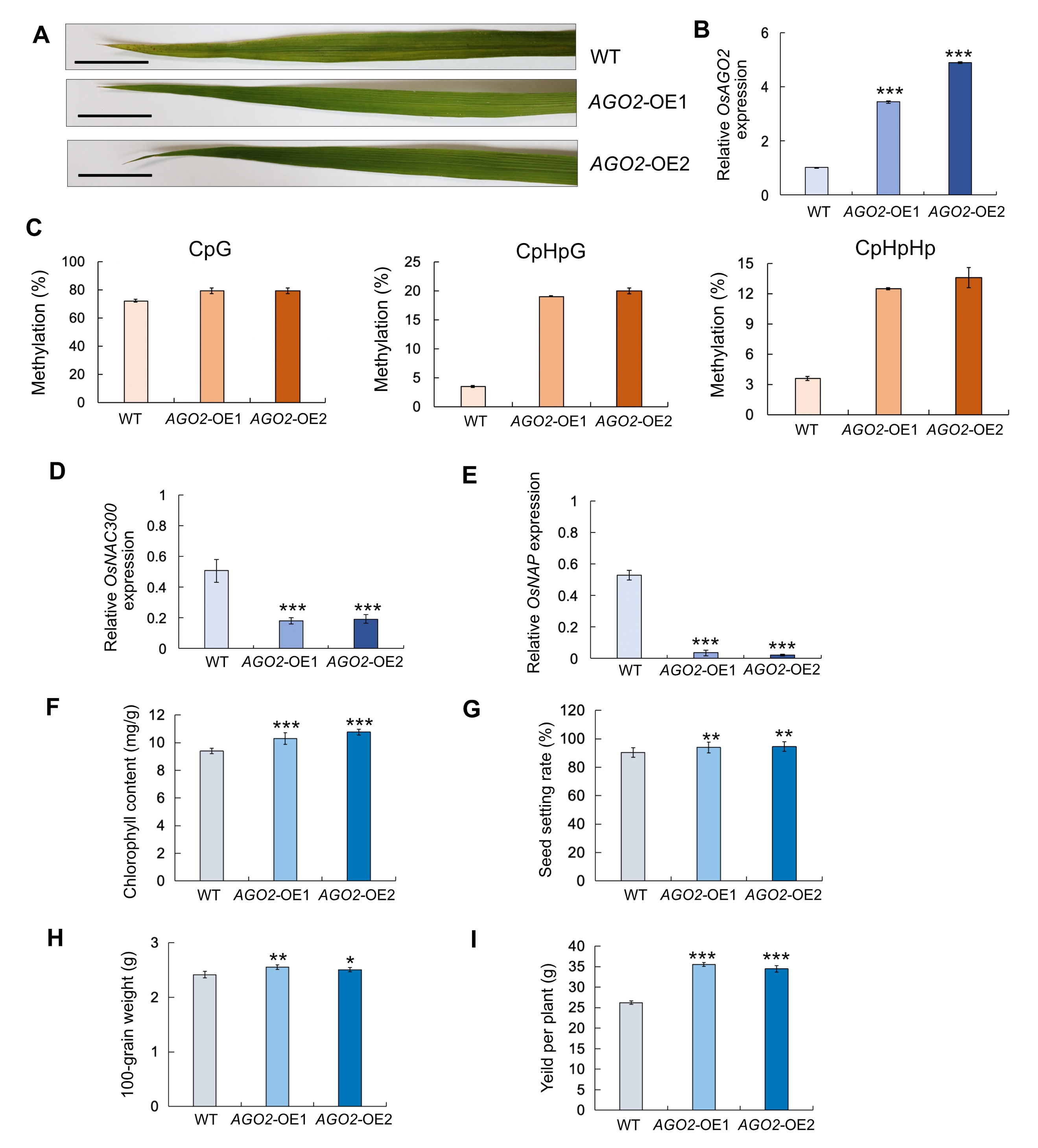


**Figure S11. The leaf characteristic analysis and agronomic traits of *AGO2* -OE plants.**

(A), The comparison of the flag leaf phenotype of WT, *AGO2-*OE1*, AGO2-*OE2 plants at the mature stage. Bars = 3 cm.

(B), Relative expression of *OsAGO2* in leaves of WT, *AGO2-*OE1*, AGO2-*OE2 plants at the mature stage. All data are presented as means ± SD of three independent replicates. ***, P < 0.001. P values were determined by Student’s *t-*test.

(C), DNA methylation analysis along the *OsNAC300* promoter region in WT, *AGO2-*OE1*,* and *AGO2-*OE2 plants by bisulfite sequencing. Sequencing data were analyzed with Kismeth software.

(D, E), Relative *OsNAC300* and *OsNAP* transcript levels in the leaves of WT, *AGO2-*OE1*, AGO2-*OE2 at the mature stage.

(F), Chlorophyll contents of WT, *AGO2-*OE1*, AGO2-*OE2 at the mature stage. All data are presented as means ± SD from three independent replicates. ***, *P* < 0.001. *P*-values were determined by Student’s *t-*test.

(G*–*I), Seed-setting rate (G), 100-grain weight (H), and grain yield per plant (I) in the WT and *AGO2*-OE lines. All data are presented as means ± SD from three independent replicates. *, 0.01 < P < 0.05. **, 0.001 < P < 0.01. ***, *P* < 0.001. *P*-values were determined by Student’s *t-*test.

**Table S1. The upregulated NAC family genes in the transcriptome data of *Osago2-1* _ wild-type (WT) and *NAC300-*OEs (overexpressions) versus wild-type (WT) (|log2 fold change|≥ 1 and Q value < 0.05).**

| Gene ID | log2FC (*Osago2-1*_WT */NAC300*-OEs_WT) | Description |
| --- | --- | --- |
| Os12g0123800 | 5.06/8.64 | OsNAC300 |
| Os07g0138200 | 6.06/7.03 | OsNAC096 |
| Os07g0683200  Os12g0610600 | 5.28/6.91  2.21/3.90 | NAC transcription factor 29  NAC, osa-miR164d |
| Os11g0126900 | 2.71/3.82 | NAC transcription factor 29 |
| Os06g0675600  Os03g0777000  Os10g0571600  Os02g0555300  Os03g0327800  Os06g0104200  Os03g0327100 | 1.96/3.83  2.48/3.50  2.98/3.27  4.63/2.72  2.18/2.51  2.47/2.22  2.51/1.99 | OsNAC1  transcription factor JUNGBRUNNEN 1  ANAC071  NAC domain-containing protein  *OsNAP*  NAC domain-containing protein  NAC domain-containing protein |
| Os02g0579000 | 1.61/1.95 | OsNAC2 |
| Os11g0154500 | 4.34/1.93 | NAC domain-containing protein |
| Os01g0104200 | 3.71/1.90 | NAC domain protein NAC1 |
| Os01g0675800 | 1.82/1.79 | NAC domain-containing protein |
| Os01g0816100 | 2.40/1.50 | NAC domain-containing protein |

**Table S2. Identified miRNAs associated with OsAGO2 by RNA immunoprecipitation—small RNA (RIP-sRNA) sequencing with an anti-Flag antibody**

| miRNA | AGO2_IP | Input | miRNA sequence | length (nt) |
| --- | --- | --- | --- | --- |
| osa-miR2863c | 142 | 6 | UUAGUAGGACUAGAAUGGGCCAAA | 24 |
| osa-miR2919 | 26 | 15 | AAGGGGGGGGGGGGAAAGA | 19 |
| osa-miR1440a | 5 | 0 | UGCUCAAAUACCACUCUCCU | 20 |
| osa-miR2925 | 3 | 0 | UGGCGGCCGCGGGCUUCGU | 19 |
| osa-miR5532 | 2 | 9 | AUGGAAUAUAUGACAAAGGUGG | 22 |
| osa-miR6256 | 2 | 0 | GUAGUACUCGGUUGUAGGUGUA | 22 |
| osa-miR414 | 2 | 0 | UCAUCCUCAUCAUCAUCGUCC | 21 |
| osa-miR5077 | 1 | 789 | GUUCGCGUCGGGUUCACCA | 19 |
| osa-miR166j-3p | 1 | 418 | UCGGACCAGGCUUCAUUCCCC | 21 |
| osa-miR166a-3p | 1 | 418 | UCGGACCAGGCUUCAUUCCCC | 21 |
| osa-miR5523 | 1 | 221 | UGAGGAGGAACAUAUUUACUAG | 22 |
| osa-miR166e-3p | 1 | 214 | UCGAACCAGGCUUCAUUCCCC | 21 |
| osa-miR2120b-3p | 1 | 2 | UUUAGUCGCGGUUGGUGUUA | 20 |
| osa-miR168b | 1 | 0 | AGGCUUGGUGCAGCUCGGGAA | 21 |
| osa-miR2865 | 1 | 0 | CUCAGCAGUCGACUGUACCGUG | 22 |
| osa-miR159a.1 | 0 | 11233 | UUUGGAUUGAAGGGAGCUCUG | 21 |
| osa-miR164a | 0 | 33 | UGGAGAAGCAGGGCACGUGCA | 21 |
| osa-miR164f | 0 | 17 | UGGAGAAGCAGGGCACGUGCA | 21 |
| osa-miR164c | 0 | 15 | UGGAGAAGCAGGGUACGUGCA | 21 |
| osa-miR164b | 0 | 3 | UGGAGAAGCAGGGCACGUGCA | 21 |
| osa-miR164e | 0 | 3 | UGGAGAAGCAGGGCACGUGAG | 21 |
| osa-miR164d | 0 | 2 | UGGAGAAGCAGGGCACGUGCU | 21 |

**Table S3. The accession numbers used in the phylogenetic analysis.**

| Protein | Accession number | Organism |
| --- | --- | --- |
| OsNAC11 | XP_025881778 | Oryza sativa |
| OsNAC096 | XP_015646514.1 | Oryza sativa |
| OsNAC2 | XP_015633924 | Oryza sativa |
| OsNAC054 | XP_015628521.1 | Oryza sativa |
| OsNAC109 | XP_015612702 | Oryza sativa |
| OsNAP | XP_015632401.1 | Oryza sativa |
| OsNAC300 | NP_001391565.1 | Oryza sativa |
| OsNAC106 | XP_015650321.1 | Oryza sativa |
| ANAC016 | NP_001322918.1 | Arabidopsis thaliana |
| ATAF1 | NP_171677.1 | Arabidopsis thaliana |
| ANAC016 | NP_001322918 | Arabidopsis thaliana |
| ANAC019 | NP_175697.1 | Arabidopsis thaliana |
| ANAC046 | NP_187056.1 | Arabidopsis thaliana |
| ANAC055 | NP_188169.1 | Arabidopsis thaliana |
| ANAC029 | NP_564966.1 | Arabidopsis thaliana |
| ANAC072 | NP_567773.1 | Arabidopsis thaliana |
| ANAC087 | NP_197328.3 | Arabidopsis thaliana |
| ANAC092 | NP_198777.1 | Arabidopsis thaliana |
| ANAC059 | NP_189546.1 | Arabidopsis thaliana |
| ANAC042 | NP_001324496.1 | Arabidopsis thaliana |
| ANAC083 | NP_196822.1 | Arabidopsis thaliana |
| ANAC032 | NP_177869.1 | Arabidopsis thaliana |
| ANAC082 | NP_001154700.1 | Arabidopsis thaliana |
| ANAC017 | NP_564440.1 | Arabidopsis thaliana |
| ANAC090 | NP_197630.1 | Arabidopsis thaliana |
| ANAC075 | NP_001329813.1 | Arabidopsis thaliana |
| ATAF2 | NP_680161.1 | Arabidopsis thaliana |

**Table S4. The genome-wide methylation levels of co-upregulated expressing genes in the transcriptome data of *Osago2* mutants versus wild-type (WT) and *NAC300*-OEs (overexpressions) versus WT. (The excel sheet)**

**Table S5. Primers used in this study.**

| Primer name | Sequence (5'-3') | Objective |
| --- | --- | --- |
| AGO2cas9-U3F | GTTGGTGGACGTGGGTATGAATC | Vector construction |
| AGO2cas9-U3R | AAACGATTCATACCCACGTCCAC | Vector construction |
| AGO2cas9-U6bF | GGCACCAGCAGCGAGGAGGATCC | Vector construction |
| AGO2cas9-U6bR | AAACGGATCCTCCTCGCTGCTGG | Vector construction |
| OEAGO2F | ATGGAGCACGAGCGCGGTGG | Vector construction |
| OEAGO2R | GATGAAGAACATGTTGTCCA | Vector Construction |
| NAC300cas9U3F | GGCAGCAACCACAGTATGCAGCCG | Vector Construction |
| NAC300cas9U3R | AAACCGGCTGCATACTGTGGTTGC | Vector Construction |
| NAC300cas9U6bF | GCCGCCGAGCAGCAACAAGCTGA | Vector Construction |
| NAC300cas9U6bR | AAACTCAGCTTGTTGCTGCTCGG | Vector Construction |
| qNAC300F | CCTAGCTTCACCTCCTTGCC | qRT-PCR |
| qNAC300R | GTTGGTTCGCATGCCAGTTG | qRT-PCR |
| NAPcas9U3F | GGCATGCTCGTGGTCGGAGAGCGG | Vector Construction |
| NAPcas9U3R | AAACCCGCTCTCCGACCACGAGCA | Vector Construction |
| NAPcas9U6bF | GGCACCTTGCAGGGATCGTCACT | Vector Construction |
| NAPcas9U6bR | AAACAGTGACGATCCCTGCAAGG | Vector Construction |
| OENAC300F | ATGGTGGAGTCTACTACATC | Vector Construction |
| OENAC300R | CTGTCGTGATATGTAATGAT | Vector Construction |
| ActinF | CACATTCCAGCAGATGTGGA | qRT-PCR |
| ActinR | GCGATAACAGCTCCTCTTGG | qRT-PCR |
| AGO2qF | TGGAGTTGGTGAATGCTGAC | qRT-PCR |
| AGO2qR | CCACTGGCAGTTAGGCTGATC | qRT-PCR |
| NAPF | CAAGAAGCCGAACGGTTC | qRT-PCR |
| NAPR | GTTAGAGTGGAGCAGCAT | qRT-PCR |
| RCCR1F | CGCATTTCCTCATGGAATTT | qRT-PCR |
| RCCR1R | CTTCTCACGCTGTTTGTCCA | qRT-PCR |
| Osh57F | ACCCTAAAGTAAATGAAGTC | qRT-PCR |
| Osh57R | CCTGCTCTTGTCTTGTTA | qRT-PCR |
| Osh36F | ATGGCGAAAAGTATGCCCGA | qRT-PCR |
| Osh36R | AGCAGGCAAGTATCCTGGTG | qRT-PCR |
| NYC1F | TGCAAGGACATTGGTTCCGA | qRT-PCR |
| NYC1R | AGAAGCGTGCTCGACTTTCA | qRT-PCR |
| NYC3F | CAGGTGCCAGAATCACCGTA | qRT-PCR |
| NYC3R | ATGGAACTGCAACTGAACCCT | qRT-PCR |
| Osl2F | ACGCTGTTCTTAGCGTGCAA | qRT-PCR |
| Osl2R | CAGTCACCACCAGTTCACCA | qRT-PCR |
| SGRF | AGGGGTGGTACAACAAGCTG | qRT-PCR |
| SGRR  Chip-P1F  Chip-P1R  Chip-P2F  Chip-P2R  Chip-P3F  Chip-P3R | GCTCCTTGCGGAAGATGTAG  ATAACTATTATTATATTATTT  CACAATTTTCAAACTCTTAACA  CCAATGCTCAAGTAGCACCT  GTAATTTGCCAGTGAACATC  ATTATCTGGCAAAACGGATTAT  GTCCTCACTCGGTTTCATTA | qRT-PCR  ChIp-qPCR  ChIp-qPCR  ChIp-qPCR  ChIp-qPCR  ChIp-qPCR  ChIp-qPCR |
| miR2863c-RT | GTCGTATCCAGTGCAGGGTCCGAGGTATTCGCACTGGATACGACTTTGGC | RT-PCR for miR2863c |
| miR2863c-qF | GCGCGTTAGTAGGACTAGAATGG | qRT-PCR |
| universal -R | AGTGCAGGGTCCGAGGTATT | qRT-PCR |
